# MYTHO is a novel regulator of skeletal muscle autophagy and integrity

**DOI:** 10.1038/s41467-023-36817-1

**Published:** 2023-03-02

**Authors:** Jean-Philippe Leduc-Gaudet, Anais Franco-Romero, Marina Cefis, Alaa Moamer, Felipe E. Broering, Giulia Milan, Roberta Sartori, Tomer Jordi Chaffer, Maude Dulac, Vincent Marcangeli, Dominique Mayaki, Laurent Huck, Anwar Shams, José A. Morais, Elise Duchesne, Hanns Lochmuller, Marco Sandri, Sabah N. A. Hussain, Gilles Gouspillou

**Affiliations:** 1 Meakins-Christie Laboratories, Translational Research in Respiratory Diseases Program, Research Institute of the McGill University Health Centre, Montréal, QC Canada; 2 Department of Critical Care, McGill University Health Centre, Montréal, QC Canada; 3 Département des Sciences de l’Activité Physique, Faculté des Sciences, Université du Québec à Montréal, Montréal, QC Canada; 4 Veneto Institute of Molecular Medicine, Padova, Italy; 5 Department of Biomedical Sciences, University of Padova, Padova, Italy; 6 Department of Pharmacology, College of Medicine, Taif University, Taif, Saudi Arabia; 7 Division of Geriatric Medicine, MUHC-Montreal General Hospital, McGill University, Montreal, QC Canada; 8 Research Institute of the McGill University Health Center, Montreal, QC Canada; 9 Département des Sciences de la Santé, Unité d’Enseignement en Physiothérapie, Université du Québec à Chicoutimi, Chicoutimi, QC Canada; 10 Children’s Hospital of Eastern Ontario Research Institute, Ottawa, ON Canada; 11 Division of Neurology, Department of Medicine, The Ottawa Hospital, Ottawa, ON Canada; 12 Brain and Mind Research Institute, University of Ottawa, Ottawa, ON Canada

**Keywords:** Macroautophagy, Diseases, Metabolism

## Abstract

Autophagy is a critical process in the regulation of muscle mass, function and integrity. The molecular mechanisms regulating autophagy are complex and still partly understood. Here, we identify and characterize a novel FoxO-dependent gene, *d230025d16rik* which we named *Mytho* (Macroautophagy and YouTH Optimizer), as a regulator of autophagy and skeletal muscle integrity in vivo. *Mytho* is significantly up-regulated in various mouse models of skeletal muscle atrophy. Short term depletion of MYTHO in mice attenuates muscle atrophy caused by fasting, denervation, cancer cachexia and sepsis. While MYTHO overexpression is sufficient to trigger muscle atrophy, MYTHO knockdown results in a progressive increase in muscle mass associated with a sustained activation of the mTORC1 signaling pathway. Prolonged MYTHO knockdown is associated with severe myopathic features, including impaired autophagy, muscle weakness, myofiber degeneration, and extensive ultrastructural defects, such as accumulation of autophagic vacuoles and tubular aggregates. Inhibition of the mTORC1 signaling pathway in mice using rapamycin treatment attenuates the myopathic phenotype triggered by MYTHO knockdown. Skeletal muscles from human patients diagnosed with myotonic dystrophy type 1 (DM1) display reduced *Mytho* expression, activation of the mTORC1 signaling pathway and impaired autophagy, raising the possibility that low *Mytho* expression might contribute to the progression of the disease. We conclude that MYTHO is a key regulator of muscle autophagy and integrity.

## Abstract (web summary)

Here, the authors identify a novel regulator of autophagy, skeletal muscle mass and integrity named MYTHO. Silencing MYTHO protects against muscle atrophy in a wide range of acute catabolic conditions, while prolonged silencing causes a severe myopathy.

## Introduction

Skeletal muscles account for ~40–50% of body mass in healthy and lean individuals and play key roles in movement, posture, thermogenesis, and whole-body metabolism. A correct balance between protein synthesis and degradation is necessary to maintain skeletal muscle mass^1^. Myofiber growth and protein synthesis are stimulated by the IGF1/AKT/mTOR pathways, whereas FoxOs transcription factors promote muscle atrophy/wasting in response to catabolic conditions such as starvation, denervation, cancer cachexia, and sepsis^2–4^. The loss of muscle mass or atrophy in these catabolic conditions involves the activation of the ubiquitin-proteasome system (UPS) and the autophagy-lysosomal pathway. Autophagy is a highly conserved quality control pathway that degrades and recycles unnecessary or damaged cellular components^5^. In the past thirty years, autophagy signaling has been extensively studied. However, the molecular machinery that regulates this multistep pathway is not yet fully elucidated^5^.

There is now substantial evidence indicating the critical role of autophagy in the regulation of skeletal muscle integrity^6,7^. For instance, muscle-selective deletion of essential autophagy genes, such as *Atg5* and *Atg7* results in the accumulation of numerous aberrant membranous structures, severe muscle weakness, and atrophy^6–9^. Defective autophagy has also been linked to several myopathies in humans^10,11^. Among the more than 250 genes that are involved in muscle disorders, several such as *Vps15*^12^, *Vcp*^13^, *Mtmr14* (also known as Jumpy)^14,15^, *Sqstm1*^16^, *Vma21*^17^, and *Lamp2*^18^, cause autophagic skeletal muscle myopathies. However, a large number of patients with myopathy remain without a definitive molecular diagnosis^19,20^. This highlights the likely contribution of novel uncharacterized genes and the need to identify and characterize novel regulators of autophagy and skeletal muscle integrity.

In skeletal muscles, FoxOs transcription factors regulate the expression of several autophagy-related genes that are necessary for autophagy induction and protein breakdown^2,21,22^. We previously showed that *FoxO1/3/4* muscle-specific knockout mice are protected from starvation- and denervation-induced muscle atrophy and weakness^2^. Additionally, the deletion of FoxOs in muscle severely impairs the induction of autophagy-related genes in response to stress. In the present study, we identified a novel FoxO-dependent Riken gene, *d230025d16rik*, which we named Macroautophagy and YouTH Optimizer (*Mytho*), as a critical regulator of autophagy and skeletal muscle integrity in vivo.

## Results

### D230025D16Rik encodes for a protein, named MYTHO, which is expressed in different tissues and transcriptionally upregulated in catabolic conditions

**Fig. 1:**
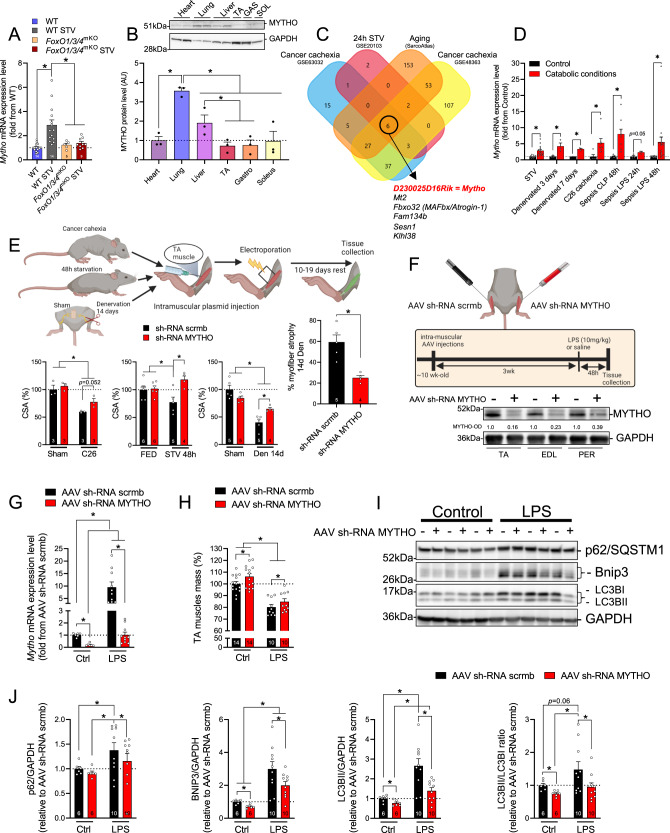
D230025D16Rik encodes for a protein, named MYTHO, which is expressed in different tissues and transcriptionally upregulated in catabolic conditions. **A** Quantification of *Mytho* mRNA expression in the tibialis anterior of fed and 24 h starved control and muscle-specific *FoxO1/3/4*^SkM-KO^ mice assessed by RT-qPCR. **B** Immunoblotting detection of MYTHO in homogenates obtained from different mouse tissues (i.e., heart, lung, liver, and muscles) of 4 months old mice (*n* = 3 mice). 50 µg of proteins were loaded for all tissues. All data in the graph are expressed relative to MYTHO content in the heart. **C** Venn diagram showing that six upregulated genes overlap among four atrophic conditions (see the “Methods” section for details). **D** Quantification of *Mytho* mRNA expression from the gastrocnemius (GAS) of starved (24 h; *n* = 14 mice in the control group; *n* = 14 mice in the starved group), denervated (*n* = 3 mice in the control group; *n* = 3 mice in the denervated group), C26 tumor-bearing (*n* = 5 mice in the control group and *n* = 7 mice in the C26 cachexia group) and septic mice (*n* = 9 mice per group for CLP experiments; *n* = 3 mice per group LPS experiments at 24 h; *n* = 15 and 17 for the control group and the sepsis LPS group at 48 h) by RT-qPCR. **E** Inhibition of MYTHO prevents muscle atrophy in mice fasted for 48 h, in C26 tumor-bearing mice, and in mouse muscles denervated for 14 days. Adult tibialis anterior (TA) muscles were transfected for 10–19 days with a sh-RNA targeting *Mytho* mRNA or a scramble sh-RNA. The cross-sectional area (CSA) of transfected fibers was quantified. **F** Schematic representation of the experimental design: TA muscles were transduced with an AAV sh-RNA scramble or an AAV sh-RNA MYTHO. The immunoblots in the lower panel confirmed the successful knockdown of MYTHO in the TA, extensor digitorum longus (EDL), and peroneus (PER) muscles 3 weeks following AAV injections. **G** Quantification of *Mytho* mRNA expression in the TA of control and septic (LPS-injected) mice by RT-qPCR (*n* = 6 mice in the control group and *n* = 10 mice in the LPS-injected group). **H** Quantification of the impact of LPS injection and MYTHO knockdown on the TA mass 48 h post-LPS or saline injection. **I**, **J** Immunoblot detection and quantification of MYTHO, p62/SQSTM1, BNIP3, and the LC3BII/LC3BI ratio in the TA 48 h post-LPS or saline injection. GAPDH was used as a loading control. All values are expressed relative to the control AAV sh-RNA scramble. The number of mice for each group is indicated within bars. Data in **A** and **B** were analyzed with one-way ANOVA and corrections for multiple comparisons were performed with the two-stage step-up method of Benjamini, Krieger, and Yekutieli (∗*p* < 0.05 and *q* < 0.1). Data in **C** were analyzed with unpaired two-tailed *t* tests (∗*p* < 0.05). Data in **E** were analyzed with two-way ANOVA and corrections for multiple comparisons were performed with the two-stage step-up method of Benjamini, Krieger, and Yekutieli (∗*p* < 0.05 and *q* < 0.1), except for data presented in the graph on the right, which was analyzed with an unpaired two-tailed *t* test (∗*p* < 0.05). Data in **G**, **H**, **J** were analyzed with two-way ANOVA, and corrections for multiple comparisons were performed with the two-stage step-up method of Benjamini, Krieger, and Yekutieli (∗*p* < 0.05 and *q* < 0.1). Data are presented as mean ± SEM (with individual data points). Detailed information on raw data, statistical tests, *p* values, and *q* values are provided in the Source Data file. The drawings in **E** and **F** were created with BioRender.com.

To identify novel FoxO-dependent putative genes regulating autophagy, we screened our published transcriptomic profiles and searched for transcripts with unknown functions that were upregulated in skeletal muscles of starved wild-type (WT) mice but unaltered in *FoxO1/3/4* muscle-specific knockout mice^2^. We subsequently decreased the number of candidates by selecting transcripts that were conserved among various species and having open reading frames (ORFs) containing autophagy-related LIRs/GIMs motifs (LC3 interaction region/GABARAP interaction motif). From this analysis, we identified the *D230025D16Rik* gene, which we named Macroautophagy and YouTH Optimizer (*Mytho*) based on its ability to regulate macroautophagy (see below) and longevity in *C. elegans* (see conference proceedings^23^). Using quantitative RT-qPCR we confirmed that *Mytho* is induced in the muscles of WT mice but not in *FoxO1/3/4*^SkM-KO^ mice in response to starvation (Fig. 1A). We also examined which factors among the FoxO family are critical and found that FoxO3 and FoxO4, but not FoxO1, were necessary for *Mytho* induction upon fasting (Fig. S1A). MYTHO, is highly conserved across species with ~ 95% amino acids sequence similarity between *C. elegans* and *H. sapiens*. The human ortholog is C16orf70 (UniProt ID: Q9BSU1), which encodes a 47.5 kDa protein. In mouse tissues, MYTHO protein is abundantly expressed in the lungs and liver and to lesser extents in the heart and various limb muscles (Fig. 1B; Fig. S1B). Notably, MYTHO protein levels were similar among muscles rich in fast-twitch glycolytic fibers (tibialis anterior and gastrocnemius) and those rich in slow-twitch oxidative fibers (soleus) (Fig. 1B). Comparisons of upregulated genes obtained from several available datasets of atrophic conditions (GSE63032, GSE20103, GSE48363, and GSE145480) revealed that muscle *Mytho* mRNA is upregulated in multiple catabolic conditions (Fig. 1C). This was confirmed by experiments conducted in-house showing that *Mytho* mRNA expression, assessed by RT-qPCR, is robustly induced in various atrophic conditions, including starvation, denervation, cancer cachexia and sepsis (Fig. 1D). Consistent with our RT-qPCR results, we confirmed that MYTHO protein level is increased in denervated skeletal muscles (Fig. S1C).

To gain insight into the physiological roles of MYTHO during catabolic conditions, we used various loss and gain of function approaches in different models known to alter autophagy and muscle mass (i.e., starvation, cancer cachexia, and sepsis). MYTHO knockdown (MYTHO-KD), achieved by electroporation of a plasmid expressing short hairpin RNA (sh-RNA) specific to MYTHO, prevented TA atrophy in acutely starved and tumor-bearing mice (Fig. 1E). MYTHO-KD also prevented in large part the myofiber atrophy triggered by 14 days of denervation (Fig. 1E). Conversely, MYTHO overexpression, achieved by either by electroporation or adeno-associated viruses (AAVs) mediated transduction, induced muscle atrophy and upregulated the expression of pro-atrophy muscle-specific ubiquitin E3 ligases *Fbxo32/Atrogin-1* and *Trim63/MuRF1*(Fig S1 D–I). Consistent with these findings, AAV-mediated MYTHO-KD over 3 weeks significantly increased muscle mass in control mice and attenuated LPS-induced muscle atrophy (Fig. 1F–H). Interestingly, MYTHO-KD attenuated the increase in *Fbxo32/atrogin-1*, but not *Trim63/MuRF1*, triggered by LPS injection (Fig. S1J, K). Similarly, MYTHO-KD attenuated the LPS-induced increase in the content of multiple autophagy proteins and markers, including p62/SQSMT1, BNIP3, and the conversion of LC3-I to LC3-II, indicating that MYTHO likely regulates autophagy (Fig. 1I, J). Consistently, mRNA expression of genes associated with the proteasome and the autophagy system, such as *Sqstm1*, *Gabarap*, *Gabarapl*, *Musa1*, *Psmb6*, *Psmb7*, *Psmb5,* and *Psmg2* were also downregulated in skeletal muscles with MYTHO-KD under basal conditions (Fig. S1L). Overall, our data indicate that MYTHO is increased in atrophic conditions and that inhibiting MYTHO can counteract atrophy during acute catabolic conditions.

### MYTHO is required for skeletal muscle autophagy

**Fig. 2:**
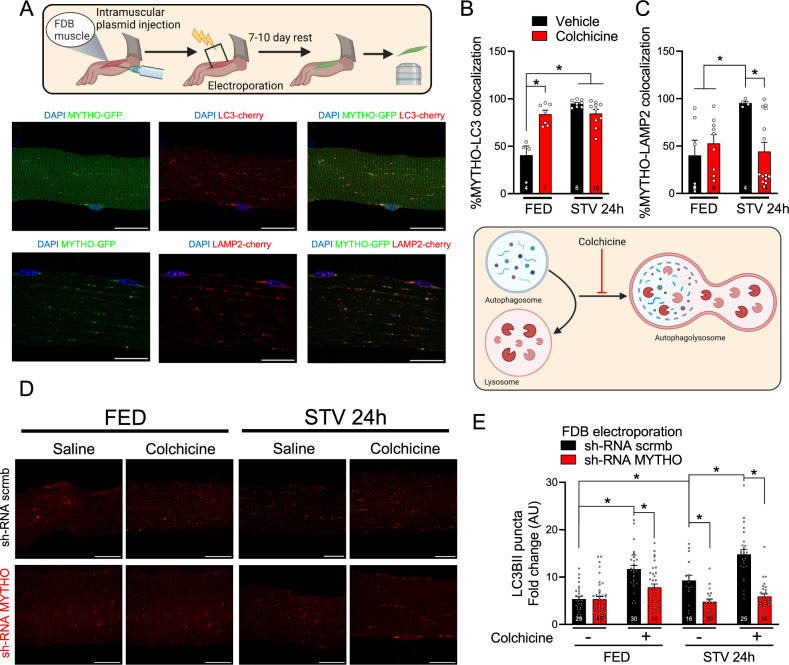
MYTHO is required for skeletal muscle autophagy. **A** Representative confocal microscope images were used for the quantification of the colocalization of MYTHO-GFP and cherry-LC3B (upper panels) or MYTHO-GFP and LAMP2-cherry (lower panels) in isolated FDB muscle fibers. Yellow dots highlight colocalization. Scale bar: 20 µm. **B** Quantification of MYTHO positive puncta that co-localizes with LC3 in single-fibers from mice treated with colchicine or vehicle in fed (FED) and 24 h starved (STV). **C** Quantification of MYTHO-positive puncta co-localizing with LAMP2-cherry in fed (FED) and 24 h starved (STV) mice that were treated with colchicine or vehicle. **D** Representative images of single FDB fibers from FED or 24 h STV mice treated with colchicine or vehicle that were co-transfected with sh-RNAs against *Mytho* or scrambled (scrmb) together with cherry-LC3B. Scale bar: 20 µm. **E** LC3 positive puncta/area were quantified in FED and 24 h STV single FDB fibers from mice treated with colchicine or vehicle for flux measurements (*n* = 3 mice per condition). Data in **B**, **C**, and **E** were analyzed with two-way ANOVA, and corrections for multiple comparisons were performed with the two-stage step-up method of Benjamini, Krieger, and Yekutieli (∗*p* < 0.05 and *q* < 0.1). Data are presented as mean ± SEM (with individual data points). Detailed information on raw data, statistical tests, *p* values, and *q* values are provided in the Source Data file. The drawings in **A** and **B** were created with BioRender.com.

To specifically address whether MYTHO plays an important role in regulating skeletal muscle autophagy, we measured MYTHO protein intracellular localization along with markers of autophagosomes (LC3B) and lysosomes (LAMP2). To achieve this goal, plasmids coding for MYTHO-green fluorescent protein (MYTHO-GFP) and LC3B-mCherry (LC3B-cherry) were electroporated in *Flexor Digitorium Brevis* (FDB) muscle of WT mice and then single muscle fibers were isolated and visualized. In isolated skeletal muscle fibers, MYTHO-GFP co-localized with LC3B-cheery (Fig. 2A, B) and, although to a lesser extent, with LAMP2 (Fig. 2A, C), confirming that MYTHO is recruited to the autophagosome. In addition, when nutrient deprivation was used to increase the autophagy flux in vivo, MYTHO localization on autophagosomes and lysosomes was enhanced (Fig. 2A–C). To assess whether localization of MYTHO-GFP is altered when the fusion of autophagosomes with lysosomes is inhibited, animals received colchicine, a drug that disrupts the microtubule networks, prior to isolation of single muscle fibers. Figure 2B, C shows that colocalization of MYTHO-GFP and LC3B-mCheery increased with colchicine treatment while colocalization of MYTHO-GFP and LAMP2-mCherry was not altered. Starvation augmented the colocalization of MYTHO-GFP and markers of autophagosomes and lysosomes; however, colchicine treatment had no effect on colocalization of MYTHO-GFP and LC3B-mCheery but significantly decreased MYTHO-GFP localization to lysosomes (Fig. 2A–C). These results suggest that MYTHO first localizes to the autophagosomes and then moves with the autophagosome to the lysosomes.

To further evaluate the involvement of MYTHO in the regulation of muscle autophagy, we investigated the impact of MYTHO-KD on the autophagic flux using colchicine treatment. In untreated WT mice, MYTHO-KD with sh-RNA oligos partially reduced the number of autophagosomes under basal conditions and blunted the increase in the number of LC3B puncta induced by starvation (Fig. 2D, E). These results indicate that MYTHO is an important regulator of skeletal muscle autophagy particularly when autophagy is increased during catabolic conditions.

### Prolonged MYTHO depletion in skeletal muscle results in excessive muscle growth, impairs muscle contractility, and induces several severe myopathy features

**Fig. 3:**
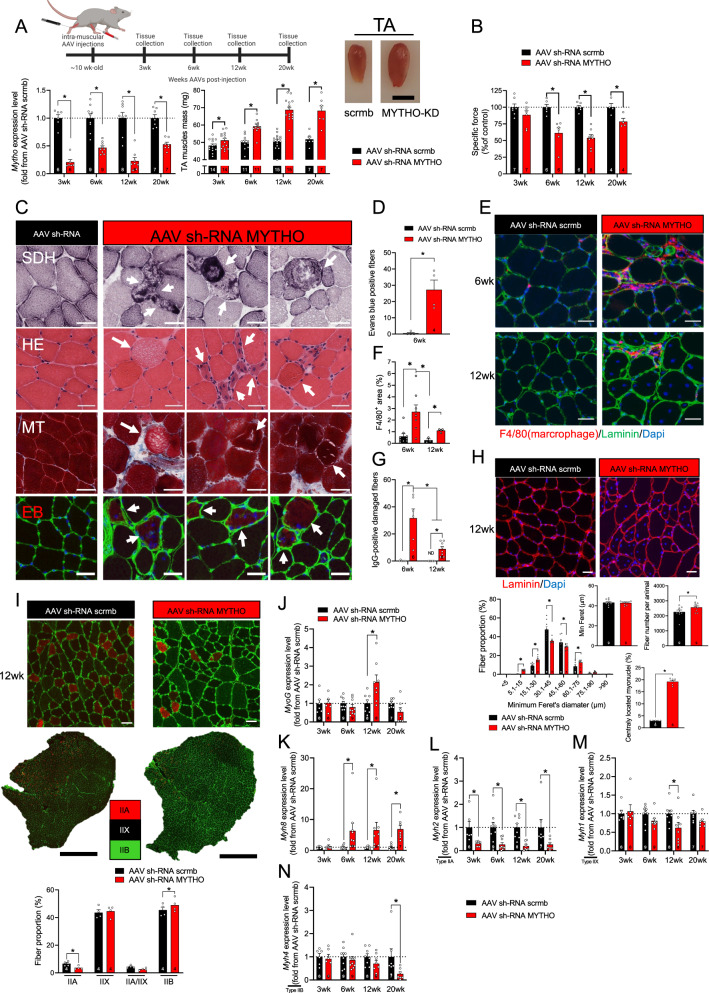
Prolonged MYTHO depletion in skeletal muscle results in excessive growth, impairs muscle contractility, and induces several severe myopathy features. **A** Upper panel: schematic representation of the experimental design. Lower left panel: quantification of *Mytho* mRNA expression in the TA at 3, 6, 12, or 20 weeks post AAV-mediated transduction (sh-RNA scramble or sh-RNA MYTHO). Lower middle panel: TA muscle mass at 3, 6, 12, or 20 weeks post AAV-mediated transduction. Lower right panel: representative image of TA muscle at 12 weeks post-AAV-mediated transduction. Scale bars = 0.5 cm. **B** Muscle-specific force measured in situ at 3, 6, 12, and 20 weeks post-injection of AAV sh-RNA scramble or AAV sh-RNA MYTHO. **C** Representative images of succinate dehydrogenase (SDH) histochemistry (upper panel), HE staining (second panel), Masson’s trichrome staining (third panel), and Evans blue staining of TA muscles at 6 weeks post AAV-mediated transduction. Arrows indicate degenerative fibers. Scale bars = 40 μm. **D** Quantification of Evans blue positive fibers in TA 6 weeks post AAV-injection **E**, **F** F4/80^+^ immunostaining and quantification of F4/80^+^ positive fibers area in TA 6 and 12 weeks post-injection of AAV sh-RNA scramble or sh-RNA MYTHO. Scale bars = 40 μm. **G** Quantification of IgG-positive fibers at 6 weeks and 12 weeks post-injection of AAV sh-RNA scramble or AAV sh-RNA MYTHO **H** Representative images of Laminin/DAPI staining (upper image) and analysis of fiber diameter, number of fibers/animal and % of centronuclear fibers (lower panels) of TA muscles transfected for 12 weeks with AAV sh-RNA scramble or AAV sh-RNA MYTHO. Scale bars = 40 μm. **I** Representative myosin heavy chain (MHC) immunolabeling and analysis of fiber type proportion and fiber diameter in TA injected with either AAV sh-RNA scramble or AAV sh-RNA MYTHO. Black scale bar = 500 µm, white scale bars = 40 µm. **J**–**N** RT-qPCR quantification of the mRNA expression of *MyoG*, *Myh8*, *Myh2*, *Myh1,* and *Myh4* in TA muscles transfected with AAV sh-RNA scramble or AAV sh-RNA MYTHO for 3, 6, 12, or 20 weeks. Sample sizes indicated in each graph represent biological replicates. Data in **A**, **B**, **D** and **I** were analyzed with paired two-tailed *t* tests (*p < 0.05). Data in **F**, **G**, **J**–**N** were analyzed with two-way ANOVA, and corrections for multiple comparisons were performed with the two-stage step-up method of Benjamini, Krieger, and Yekutieli (∗*p* < 0.05 and *q* < 0.1). The min ferret diameter distribution in **H** was analyzed with two-way ANOVA and corrections for multiple comparisons were performed with the two-stage step-up method of Benjamini, Krieger, and Yekutieli (∗*p* < 0.05 and *q* < 0.1). All other comparisons in **H** were analyzed with paired two-tailed *t* tests (*p < 0.05). Data are presented as mean ± SEM (with individual data points). Detailed information on raw data, statistical tests, *p* values, and *q* values are provided in the Source Data file. The drawing in **A** was created with BioRender.com.

To further explore the physiological role of MYTHO in controlling skeletal muscle mass and function, we tested the impact of MYTHO-KD at various time points. Consistent with previous observations, AAV-mediated MYTHO-KD gradually increased skeletal muscle mass at 3-, 6-, 12-, and 20 weeks post AAV transduction (Fig. 3A). To define the physiological and functional impact of MYTHO-KD on skeletal muscles, the contractile properties of the TA were assessed at 3-, 6-, 12-, and 20 weeks post AAV-mediated transduction. At 3 weeks following AAV injection, no significant change in specific force was seen in muscles with MYTHO-KD (Fig. 3B and Fig. S2A). However, at 6, 12, and 20 weeks following AAV injection, the TA-specific force was significantly lower in muscles with MYTHO-KD (Fig. 3B and Fig. S2B–D). These data demonstrate that the muscle mass gained in response to prolonged MYTHO-KD is largely non-functional.

To gain insight into potential mechanisms through which MYTHO-KD affects muscle contractility, we performed a histological examination of muscle sections. Three weeks of MYTHO-KD triggered no major histological abnormalities except for the very rare appearance of ragged blue fibers (Fig. S2G, H). Six weeks of MYHTO-KD resulted in several histological abnormalities such as ragged blue fibers, fibers with central nuclei, inflammatory cells infiltration, myofiber necrosis, and small-diameter regenerating fibers (Fig. 3C and Fig. S2E, F). We next assessed myofiber membrane integrity using the Evans blue dye (EBD) uptake. Six weeks of MYHO-KD was associated with an increased number of EBD-positive fibers (Fig. 3C, D). Given that macrophages play a critical role in the early phase of muscle regeneration from necrotic fiber injury^24^, muscle sections were stained for F4/80, a macrophage-specific marker. We also stained muscle sections for mouse IgG-positive fibers as an index of cell membrane damage. Six weeks of MYTHO-KD, and to a lesser extent twelve weeks of MYTHO-KD, increased macrophage infiltration (F4/80+ area) in several muscle fibers and elicited the appearance of IgG-positive fibers (Fig. 3F, G and Fig. S2I). Taken together, our data clearly indicate that prolonged MYTHO-KD results in the appearance of severe myopathic features. These data also highlight that MYTHO-KD triggers an inflammatory response that peaks at 6 weeks post-AAV transduction.

To further investigate the impact of MYTHO-KD on skeletal muscle health, we next immunostained muscle cross-sections for laminin, a protein of the basal membrane, and nuclei (using DAPI) and showed that 12 weeks of MYTHO-KD results in abnormal fiber size variation, an increase in the total number of myofibers and an increase in the number of fiber with central nuclei in the TA (Fig. 3H). The rearrangement of myonuclei in skeletal muscle is a typical sign of various myopathies with or without degeneration/regeneration^25^. We found that the abnormal fiber size variation at 12 weeks after MYTHO-KD was accompanied by a shift in fiber types characterized by an ~8% increase in the proportion of type IIB fibers and a ~45% decrease in the proportion of type IIA fibers (Fig. 3I and Fig. S2J). This shift in fiber types triggered by MYTHO-KD was associated with alterations in the expression of genes coding for various myosin heavy chain (MHC) isoforms and several myogenic markers of regeneration (Fig. 3J–N). Indeed, the expressions of *Myog* (which encodes myogenin) and perinatal MHC isoform (*Mhy8*) were significantly increased at various time points after MYTHO-KD, whereas *Myh2* (a gene encoding for type IIA MHC) was significantly downregulated at 3, 6, 12 and 20 weeks after AAV transduction. These results further strengthen that prolonged MYTHO-KD causes various myopathic features.

**Fig. 4:**
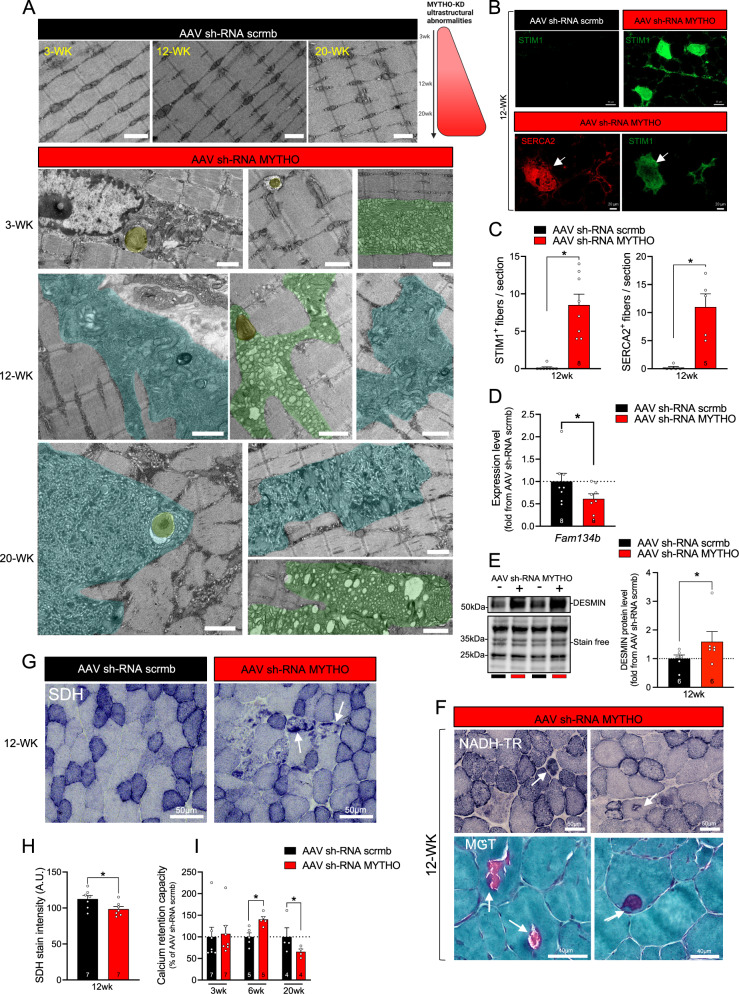
MYTHO depletion induces severe ultrastuctural anormalities. **A** Electron micrographs of GAS muscles at 3, 12, and 20 weeks post AAV-mediated transduction of sh-RNA scramble or sh-RNA MYTHO. Ultrastructural analysis reveals abundant tubular aggregates and other cytoplasmic vacuolar material. These ultrastructural defects induced by MYTHO knockdown were observed in all samples examined (*n* = 4 mice per group). Scale bars = 1 μm. **B**–**H** is from TA muscles at 12 weeks post AAV-mediated transduction. **B**, **C** Representative SERCA2 (red) and STIM1 (green) immunolabeling and analysis of STIM1 positive fibers in TA muscles. Arrows indicate that SERCA2-positive myofibers are the same as STIM1-positive fibers. **D** Quantification by RT-qPCR of *Fam134b* mRNA expression in TA muscles. Data were normalized by the geometric mean of *18* *S*, *β-actin,* and *cyclophilin*. **E** Immunoblot detection and corresponding quantification of DESMIN content. **F** NADH-TR and modified Gomori trichrome (MGT) staining reveal core-like lesions and ragged-red myofibers in muscles with MYTHO knockdown. These defects induced by MYTHO knockdown were observed in all samples examined (*n* = 4 mice). **G**, **H** Representative images of SDH staining and quantification of SDH intensity. Arrows indicate ragged blue/red fibers. **I** Calcium retention capacity and time to mPTP (mitochondrial permeability transition pore) opening assessed in permeabilized myofibers from GAS muscles at 3, 6, and 20 weeks post AAV-mediated transduction. The number of mice in each group is displayed in each bar. Data in **C**, **D**, and **H** were analyzed with paired two-tailed *t* tests (*p < 0.05). Data in **I** were analyzed with two-way ANOVA and corrections for multiple comparisons were performed with the two-stage step-up method of Benjamini, Krieger, and Yekutieli (∗*p* < 0.05 and *q* < 0.1). Data are presented as mean ± SEM (with individual data points). Detailed information on raw data, statistical tests, *p* values, and *q* values are provided in the Source Data file.

The clear myopathic phenotype triggered by prolonged MYTHO-KD was further confirmed by the examination of muscle samples using electron microscopy. As shown in Fig. 4A, we found striking ultrastructural abnormalities in MYTHO-KD muscle, including dilated sarcoplasmic reticulum (SR), lamellar bodies, swollen mitochondria, glycogen accumulation, a large accumulation of autophagic material and tubular aggregates (Fig. 4A). Tubular aggregates are usually associated with several skeletal muscle disorders^26^ and are believed to derive from the expansion of the SR due to altered Ca^2+^ homeostasis^27^. Consistent with this notion, we observed an increase in the number of STIM1 and SERCA2 positive fibers in muscles with MYTHO-KD (Fig. 4B, C). As shown in Fig. 4D, MYTHO depletion decreased the expression of *Fam134b*, an important ER-phagy receptor. Similar to centronuclear and myofibrillar myopathies^28,29^, we observed an accumulation of desmin protein in muscles with MYTHO-KD (Fig. 4E). Interestingly, NADH-TR staining of MYTHO-KD TA muscle reveals core-like lesions that resembled central core disease (Fig. 4F). To assess whether MYTHO-KD alters mitochondrial enzyme expression, we stained muscle section for the activity of succinate dehydrogenase (SDH). The overall intensity of SDH activity staining was significantly lower in muscles with MYTHO-KD relative to those with intact MYTHO expression (Fig. 4G, H). Moreover, several ragged SDH-positive fibers were detected in muscles with MYTHO-KD (Fig. 4G). In saponin-permeabilized myofibers, measurements of mitochondrial Ca^2+^ retention capacity revealed biphasic changes with an increase after six weeks and a decrease after twenty weeks of MYTHO-KD (Fig. 4I). Despite these changes in mitochondrial Ca^2+^ retention capacity, muscle mitochondrial respiration and H_2_O_2_ production remained unchanged after different periods of MYTHO-KD, suggesting that intrinsic mitochondrial dysfunction may not be involved in the myopathic phenotype triggered by MYTHO-KD (Fig. S3A–C). Collectively, our data show that MYTHO depletion in skeletal muscle caused a severe myopathy with a wide spectrum of ultrastructural pathological changes, including tubular aggregates and the presence of numerous vacuoles containing amorphous material.

### MYTHO-KD triggers activation of growth signaling and impaired autophagy

**Fig. 5:**
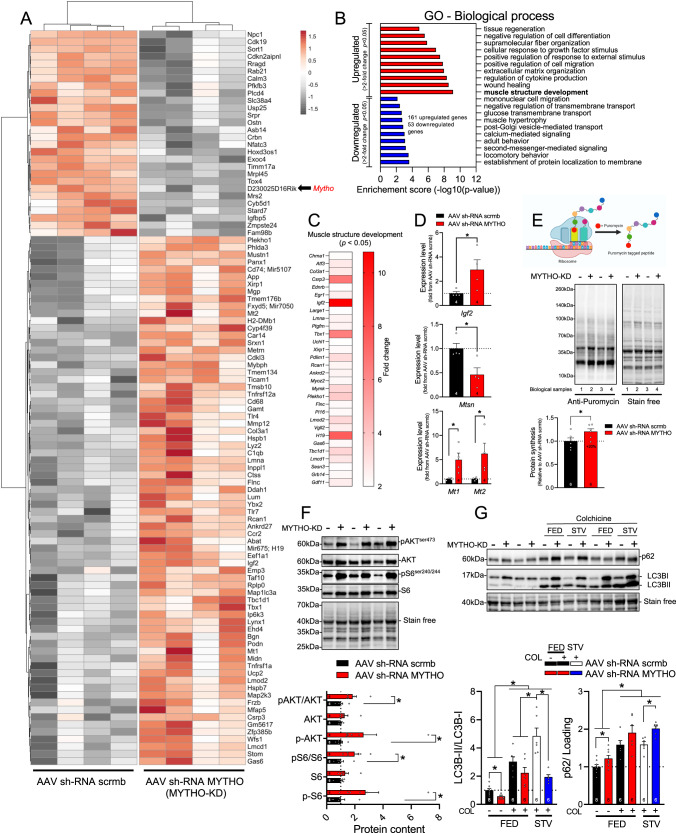
MYTHO depletion activates growth signaling. **A** Heatmap from microarray showing Gastrocnemius (GAS) muscle gene expression signatures at 12 weeks post AAV-mediated transduction of sh-RNA scramble or sh-RNA MYTHO. Colors indicate relative expression levels; red indicates high expression and gray indicates low expression. **B** Top ten upregulated (red) and downregulated (blue) pathways upon MYTHO knockdown (MYTHO-KD) as identified through GO enrichment analysis. **C** Heatmap highlighting selected upregulated genes from the GO annotation “skeletal muscle growth” or from previously published research. **D** RT-qPCR quantification of the impact of MYTHO knockdown on *Igf2* and *Mstn, Mt1, and Mt2* mRNA expression in the GAS. *β-Actin* was used as a reference gene. **E**–**G** are from TA muscles at 12 weeks post AAV-mediated transduction. **E** Immunoblot detection and quantification of puromycin incorporation in muscles (in vivo SUnSET technique). **F** Immunoblot detection and quantification of p-AKT, AKT, p-S6, and S6. **G** Immunoblot detection and quantification of p62 and LC3II/I accumulation in TA muscle from fed or starved (48 h) mice treated with colchicine or vehicle for flux measurements. Stain-free images were used to normalize protein contents. The number of mice for each group is indicated within bars. Data in **D** were analyzed with paired one-tailed *t* tests (*p < 0.05). Data in **E** were analyzed with paired two-tailed *t* tests (*p < 0.05). Data in **F** were analyzed with two-way ANOVA and corrections for multiple comparisons were performed with the two-stage step-up method of Benjamini, Krieger, and Yekutieli (∗*p* < 0.05 and *q* < 0.1). Data in **G** were analyzed with either paired two-tailed *t* tests to compare the impact of MYTHO-KD in each group or unpaired two-tailed *t* tests for all other comparisons (∗*p* < 0.05). Data are presented as mean ± SEM (with individual data points). Detailed information on raw data, statistical tests, *p* values, and *q* values are provided in the Source Data file. The drawing in **E** was created with BioRender.com.

To address the mechanisms underlying muscle hypertrophy and myopathic features induced by MYTHO-KD, microarray analyses were performed on GAS muscle samples obtained three- and twelve weeks post MYTHO-KD (full list of differentially expressed (DE) genes is provided in the Source Data File). As shown in Fig. 5A, which displays a heatmap of the top 100 most robustly regulated genes (>2-fold change and *p* < 0.05) detected after 12 weeks of MYTHO-KD. Prolonged MYTHO-KD altered the expression of several genes involved in skeletal muscle growth and development such as insulin-like growth factor 2 (*Igf2*), musculoskeletal embryonic nuclear protein 1 (*Mustn1*), long non-coding RNA *H19*, myosin binding protein H (*Mybph*), and LIM and cysteine-rich domains 1 (*Lmcd1*) (Fig. 5A). Gene set enrichment analyses (GSEA) of 161 upregulated and 53 downregulated genes (>2-fold change and *p* < 0.05) showed marked enrichment of gene sets involved in cellular response of growth factor stimulus, muscle structure development, wound healing and regulation of cytokine production (Fig. 5B, C). We verified using RT-qPCR that twelve weeks of MYTHO-KD increased expression of *Igf2* and metallothioneins *Mt1* and *Mt2*, and decreased expression of *Mtsn* (Fig. 5D). Similar to gene expression alterations by twelve weeks of MYTHO-KD, transcriptome analyses of muscles with three weeks of MYTHO-KD revealed upregulation of several genes involved in skeletal muscle structure development (Fig. S4A–C).

Considering the progressive hypertrophy triggered by MYTHO-KD, combined with the increased expression of genes involved in growth signaling, we next investigated whether MYTHO-KD altered muscle protein synthesis in vivo by performing puromycin incorporation analysis^30^. Twelve weeks of MYTHO-KD elicited an increase in muscle protein synthesis, as indicated by the rise in puromycin-labeled peptide levels (Fig. 5E). A similar trend was observed in muscles with three weeks of MYTHO-KD (Fig. S4D). We also observed that MYTHO-KD is associated with increased phosphorylation of AKT and S6 (downstream target of mammalian target of rapamycin complex 1 (mTORC1)) (Fig. 5F). These results indicate that depletion of MYTHO in skeletal muscle results in sustained activation of growth signaling pathways.

To assess whether increased muscle mass associated with MYTHO-KD was mediated in part through inhibition of protein degradation, we assessed the activities of autophagy by measuring autophagic flux. Confirming the key role of MYTHO in regulating autophagy (Fig. 2), we found that twelve weeks of MYTHO-KD impaired muscle autophagy, as evidenced by an accumulation of p62/SQSTM1 and LC3BI, and decreased LC3BII/LC3BI ratios (Fig. 5G). The increase in muscle autophagy in response to acute starvation (as assessed by the change in LC3BII/I ratio in starved and colchicine-treated mice) was blunted in muscles with MYTHO-KD (Fig. 5G), indicating that long-term MYTHO-KD resulted in sustained impairment of autophagy. Further strengthening the role played by MYTHO in autophagy, marked accumulation of p62/SQSTM1, ATG7, and LC3BI are still seen in muscles with MYTHO-KD 20 weeks post AAV injection (Fig. S5A–E).

### Autophagy is dispensable for MYTHO depletion to induce a myopathic phenotype

**Fig. 6:**
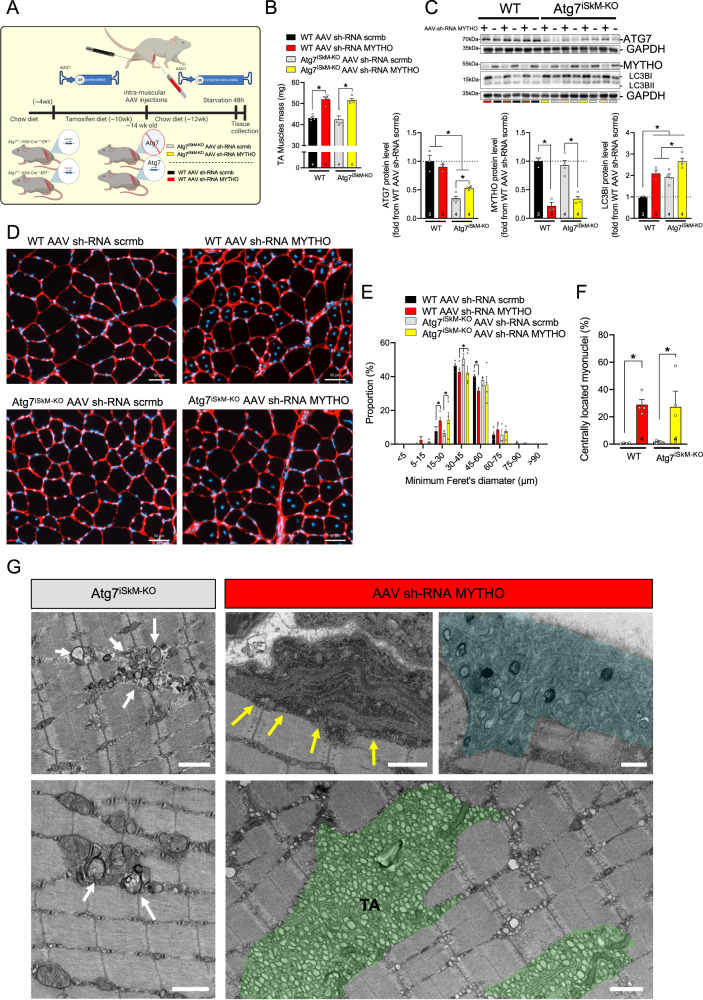
Autophagy is dispensable for MYTHO depletion to induce a myopathic phenotype. **A** Schematic representation of the experimental design. **B** TA muscle mass from WT and *Atg7*^iSkM-KO^ mice at 12 weeks post AAV sh-RNA scramble or AAV sh-RNA MYTHO injections. **C** Immunoblot detection of various autophagic proteins and corresponding quantifications performed on TA muscle samples from WT and *Atg7*^iSkM-KO^ mice at 12 weeks post AAV sh-RNA scramble or AAV sh-RNA MYTHO injections. **D**–**F** Representative images of laminin and DAPI staining and analysis of muscle fiber diameter and a number of fibers with centralized nuclei 12 weeks post AAV injections. Scale bars in **D** = 50 μm. **G** Representative images of electron micrographs of the white GAS muscles from *Atg7*^iSkM-KO^ mice and muscles from WT mice injected with AAV sh-RNA MYTHO showing more severe ultrastructural abnormalities in muscles with MYTHO-KD (*n* = 2 *Atg7*^iSkM-KO^ mice and *n* = 4 mice with MYTHO-KD). White arrows indicate abnormal membrane structures. Yellow arrows indicate electron-dense granular and filamentous aggregates. The area highlighted in blue presents multiple ultrastructural defects including lamellar bodies. TA indicates the accumulation of tubular aggregates (colored in green). Scale bars = 1 μm. The number of mice for each group is indicated within bars. Data in **B**, **C**, **E**, and **F** were analyzed with two-way ANOVA, and corrections for multiple comparisons were performed with the two-stage step-up method of Benjamini, Krieger, and Yekutieli (∗*p* < 0.05 and *q* < 0.1). Data are presented as mean ± SEM (with individual data points). Detailed information on raw data, statistical tests, *p* values, and *q* values are provided in the Source Data file. **A** was created with BioRender.com.

Considering that MYTHO plays a key role in the autophagic process in vivo (Fig. 2), we next investigated whether myopathic features associated with MYTHO-KD are due to the inhibition of autophagosome formation. To test this possibility, we elicited MYTHO-KD in skeletal muscles of muscle-specific inducible *Atg7* knockout mice (muscle *Atg7*^iSkM-KO^)^6,7^ (Fig. 6A). Deletion of *Atg7* in skeletal muscles resulted in the prevention of autophagosome formation (Fig. 6C). Acute treatment with tamoxifen greatly reduced Atg7 content and autophagy in *Atg7*^iSkM-KO^ mice, as revealed by the accumulation of LC3BI. Knocking down MYTHO for 12 weeks increased skeletal muscle mass to a similar extent in *Atg7*^iSkM-KO^ and WT mice (Fig. 6B), suggesting that MYTHO has a broader role than being an autophagy regulator. The depletion of MYTHO and ATG7 in skeletal muscle resulted in a marked accumulation of LC3BI which was more pronounced in MYTHO-KD muscles obtained from *Atg7*^iSkM-KO^ mice relative to WT mice (Fig. 6C).

Importantly, MYTHO-KD resulted in a similar increase in the proportion of centrally located myonuclei in muscles of WT and *Atg7*^iSkM-KO^ mice, while *Atg7* deletion alone had no impact on this parameter (Fig. 6D–F). Although electron microscopy confirmed the accumulation of membranous structures in muscles of *Atg7*^iSkM-KO^ mice, those ultrastructural abnormalities were clearly distinct and less severe than those observed in muscles with MYTHO-KD (Fig. 6G). These results suggest that disruption of autophagy is one of the systems, but not the unique one, controlled by MYTHO and involved in the myopathic phenotype.

### Rapamycin treatment ameliorates MYTHO-KD-induced myopathy

**Fig. 7:**
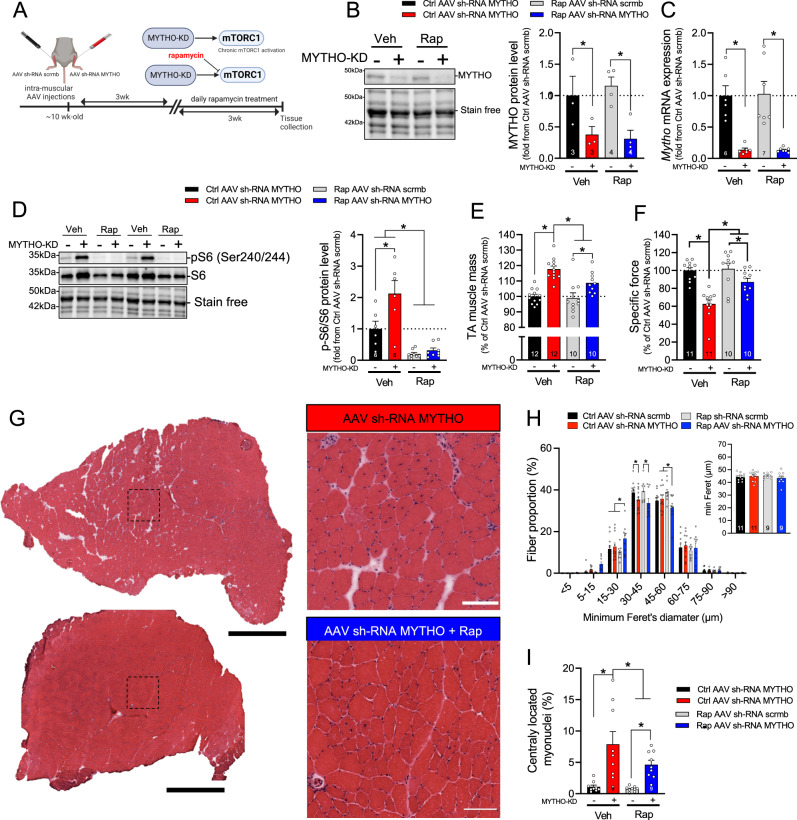
Rapamycin treatment ameliorates MYTHO knockdown (MYTHO-KD) induced myopathy. **A** Schematic representation of the experimental design. **B** Immunoblot detection and quantification of MYTHO in TA muscles from mice treated for 3 weeks with rapamycin (Rap) or vehicle at 3 weeks post AAV-mediated transduction. **C** Quantification of *Mytho* mRNA expression in muscle assessed by RT-qPCR. Data were normalized by the geometric mean of *18* *S*, *β-Actin,* and *Cyclophilin*. **D** Immunoblots detection and quantification of pS6 and total S6 in TA muscles demonstrating mTORC1 inhibition in mice treated with rapamycin. **E** TA muscle mass from mice treated with vehicle or rapamycin 6 weeks post AAV-mediated transduction. **F** in situ assessment of muscle-specific force in AAV sh-RNA scramble and AAV sh-RNA MYTHO injected TA muscles from mice treated with vehicle or rapamycin. **G** Representative H&E staining, **H** corresponding analysis of muscle fiber diameter and **I** proportion of fibers with centralized nuclei in AAV sh-RNA scramble and AAV sh-RNA MYTHO TA muscles from mice treated for 3 weeks with rapamycin or vehicle at 3 weeks post AAV-mediated transduction. Large scale bars = 200 µm, inset scale bar = 100 µm. The number of mice for each group is indicated within bars. Data in **B**–**F**, **H**, **I** were analyzed with two-way ANOVA, and corrections for multiple comparisons were performed with the two-stage step-up method of Benjamini, Krieger, and Yekutieli (∗*p* < 0.05 and *q* < 0.1). Data are presented as mean ± SEM (with individual data points). Detailed information on raw data, statistical tests, *p* values, and *q* values are provided in the Source Data file. **A** was created with BioRender.com.

Since MYTHO-KD results in a chronic hyperactivation of the mTORC1 pathway, we reasoned that blocking this pathway using rapamycin treatment would prevent or at least attenuate the myopathic phenotype triggered by MYTHO-KD. To test this hypothesis, MYTHO was first KD for three weeks. Mice then received daily injections of rapamycin or vehicle for another three weeks. Muscles were then examined after six weeks of MYTHO-KD, at a point where central nuclei, prominent inflammatory cell infiltration, and signs of muscle degeneration/regeneration are clearly present (Fig. 3C-G). As shown in Fig. 7B-C, MYTHO-KD was successful in the skeletal muscles of both vehicle-treated and rapamycin-treated mice. As expected, MYTHO-deficient muscle showed increased phosphorylation of S6^Ser240/244^, whereas mice treated with rapamycin showed a decrease in the phosphorylation of S6^Ser240/244^ (Fig. 7D). As expected, MYTHO-KD increased muscle mass (~18% increase) in mice that received vehicle treatment. However, this impact of MYTHO-KD was blunted in rapamycin-treated mice (only ~9% increase) (Fig. 7E). Similarly, peak tetanic isometric TA specific force decreased by ~38% in response to MYTHO-KD in animals which received vehicle while this decrease in peak force was only 13% in animals which received rapamycin (Fig. 7F). Rapamycin treatment also attenuated the proportion of fibers with central nuclei but had no major effect on overall minimum Feret diameters of muscle fibers upon MYTHO-KD (Fig. 7G-I). These data indicate that inhibiting mTORC1 with rapamycin partly rescues the deleterious impact of MYTHO-KD on muscle function and integrity.

### MYTHO is downregulated in myotonic dystrophy type 1

MYTHO-KD-induced myopathy shares multiple features with myotonic dystrophy type 1 (DM1) including muscle weakness, fiber size variability, centralized myonuclei, and multiple EM ultrastructural abnormalities^31–34^. Similar to DM1^31,32^, we also found AMPK signaling is repressed, while mTORC1 signaling is increased in MYTHO-KD muscles (Fig. S5F–H). Interestingly, RNA-seq data of skeletal muscle biopsies derived from healthy and DM1 individuals indicated that DM1 disease severity is associated with lower *Mytho* expression (Fig. S5I). We confirmed by RT-qPCR that *Mytho* expression is downregulated in muscle biopsies from DM1 patients compared with age-matched healthy controls (Fig. S5J). Consistent with data previously reported^32^, we found that phosphorylation levels of S6 was elevated in DM1 patients, whereas LC3II and p62 levels were reduced (Fig. S5K, L). Taken together with our current data, these findings raise the possibility that low *Mytho* expression, aberrant mTORC1 activation, and dysregulated autophagy signaling might contribute to the progression of DM1 and may play a role in other myopathies.

## Discussion

In the present study, we show that *Mytho* is a FoxO-dependent gene that regulates skeletal muscle autophagy in vivo and whose expression is upregulated in various catabolic conditions that trigger muscle atrophy. We also show that short-term knockdown of MYTHO attenuated muscle atrophy caused by starvation, denervation, cancer cachexia, and sepsis. While MYTHO overexpression was sufficient to trigger muscle atrophy, MYTHO knockdown resulted in a progressive increase in muscle mass associated with sustained activation of the mTORC1 signaling pathway. Long-term MYTHO knockdown leads to a progressive severe myopathic phenotype, characterized by impaired autophagy, muscle weakness, myofiber degeneration, and ultrastructural abnormalities, including accumulation of membranous structures, aberrant autophagic vacuoles, and tubular aggregates. Our study provides evidence that the myopathic phenotype induced by MYTHO-KD can be rescued, at least in part, by inhibiting the mTORC1 signaling pathway.

One of the main findings of the present study is that MYTHO is required for proper autophagic function in skeletal muscle in vivo. We show that MYTHO first localizes on autophagosomes and then is carried to autophagolysosomes. Depletion of MYTHO in skeletal muscle is sufficient to inhibit the basal level of autophagy and especially to blunt the increase in the autophagic flux in response to starvation, one of the most potent stimuli to activate autophagy in muscle cells^35^. Importantly, our time course experiments performed over 20 weeks revealed that MYTHO-KD consistently increases LC3BI levels, suggesting that MYTHO plays a key role in the early phases of autophagy, most likely during phagophore formation and/or autophagosome formation. Consistent with the idea that MYTHO plays a key role in autophagy, prior observations from two independent screens in humans have highlighted a possible interaction between C16orf70 (MYTHO) and WIPI2^36,37^, an essential protein for autophagosome formation from phagophores^38^. Very recently, and while our experiments were well underway, Kojima et al. reported data in HeLa cells indicating that C16orf70 (a.k.a. MYTHO) interacts with BCAS3 (microtubule-associated cell migration factor) to form a complex that associates with the phagophore assembly site^39^. These authors did not observe any impairment in mitophagy in *BCAS3*^−/−^ and *C16orf70*^−/−^ cells, leading them to suggest that C16orf70 and BCAS3 are likely regulatory proteins that fine-tune the autophagic activity^39^. Our study not only confirms the importance of MYTHO in the autophagic process in mammals, but also indicates that MYTHO is required for proper muscle autophagic function in vivo. These findings are in accordance with a recent study conducted in *Dictyostelium* cells that demonstrated that the loss of *KinkyA* and DDB_G0272949, homologs of mammalian C16orf70 and BCAS3, respectively, impair autophagosome formation^40^.

Another important finding of the present study is that short-term MYTHO-KD confers protection against fiber atrophy induced by starvation, denervation, cancer cachexia, and sepsis, while MYTHO overexpression is sufficient to trigger muscle atrophy. This apparent protective impact of MYTHO-KD in various catabolic conditions shares similarities with the protection seen upon VCP knockdown (VCP being a protein playing roles in autophagy and lysosomal homeostasis) which also increases muscle mass and confers protection against denervation and fasting-induced muscle wasting^41^. However, the protection against muscle atrophy conferred by MYTHO-KD differs from what was reported in *Atg7*^iSkM-KO^ mice where autophagosome formation is completely prevented. Indeed, *Atg7* ablation results in muscle atrophy and worsening of denervation- and acute starvation-induced muscle wasting^6,7^. These findings indicate that MYTHO likely plays roles either in autophagy enhancement in catabolic conditions or outside the autophagy system.

In this study, we provide compelling evidence indicating that long-term MYTHO-KD results in a severe myopathic phenotype characterized by a progressive increase in muscle mass, an increase in total fiber number, inflammatory cell infiltration, signs of muscle degeneration and regeneration, glycogen accumulation, mitochondrial swelling, severe accumulation of membranous like structures and tubular aggregates. This myopathic features triggered by MYTHO-KD have been detected in several myopathies including muscular dystrophies and centronuclear/myotubular myopathies^42^. Tubular aggregates are abnormalities characterized by the accumulation of densely packed tubules of variable forms and sizes and are believed to derive from the expansion of the sarcoplasmic reticulum (SR) due to altered Ca^2+^ homeostasis^27^. Tubular aggregates are found in several skeletal muscle disorders, including myotonic dystrophy^33^, myopathies resulting from STIM1 (stromal interaction molecule 1), ORAI1 (calcium release-activated calcium modulator (1) mutations^43,44^, and dystrophies associated with mutations in glycosylation-related genes^26^. Several proteins involved in the storage and update of Ca^2+^ such as SERCA1 (sarco/endoplasmic reticulum Ca^2+^ ATPase), STIM1, RYR1 (ryanodine receptor 1), and sarcolumenin were identified as components of tubular aggregates^45^. We found that muscles with MYTHO-KD have fibers with aberrant STIM1 staining and mitochondrial Ca^2+^ retention dysregulation, suggesting that MYTHO-KD alters muscle fiber Ca^2+^ homeostasis. It should be noted that the pathological phenotype associated with MYTHO-KD including the presence of fibers with central nuclei and tubular aggregates is much more severe than that observed in Atg7-deficient muscles.

Whether dysfunctional or mutated MYTHO plays a role in human muscular diseases is currently unknown. One study using data mining to identify and rank candidate genes associated with myopathies concluded that C16ORF70 (MYTHO) is a candidate gene for autophagic vacuolar myopathy^46^. In the current study, we measured MYTHO expression in muscle biopsies of patients with DM1^32^, a neuromuscular genetic disease sharing several features with muscles with MYTHO-KD. Our results indicate that *Mytho* expression is significantly decreased in the muscles of patients with severe DM1 raising the possibility that the downregulation of *Mytho* may play a role in the pathophysiology of DM1. Interestingly, it has been reported that the methylation of C16ORF70 is dysregulated in patients with Schizophrenia^47^, suggesting that dysregulation of *Mytho* expression may also contribute to the pathogenesis of diseases affecting organs other than skeletal muscles.

Another important finding from the present study is the demonstration that the hypertrophic and myopathic phenotype induced by MYTHO-KD is caused, at least in part, by a sustained hyperactivation of the mTORC1 pathway. Indeed, rapamycin treatment, an inhibitor of mTORC1, partly rescued the impact of MYTHO-KD on muscle mass, strength, and markers of muscle degeneration/regeneration. Hyperactivation of mTORC1, which is known to trigger myopathy^48,49^, is emerging as a common feature in all kinds of muscles disorders, including sarcopenia^50^, myofibrillar myopathy^29^, laminopathies^51^, dystroglycanopathies^52^, and DM1^32^. Interestingly, rapamycin treatment was also shown to confer protection in the setting of mitochondrial myopathies^53^. Our results, therefore, strengthen available literature positioning partial inhibition of mTORC1 as a potential strategy to improve patient outcomes in various muscular diseases^32,50–52,54^. The mechanism linking MYTHO-KD to sustained hyperactivation of the mTORC1 pathway and growth signaling pathways will require further studies. However, a potential candidate mechanism is MYTHO-KD-induced dysregulation of secreted factors such as IGF2 and myostatin. Indeed, we observed significant induction of *Igf2* gene expression and decreased myostatin expression in muscles with prolonged MYTHO-KD. The IGF2 receptor was previously reported to increase in dystrophic muscles and blockage of IGF2R ameliorates myopathy in *mdx* mice, a model of Duchenne muscular dystrophy^55^. Decreased expression of myostatin in skeletal muscles has also been reported in mouse and dog models of myotubular myopathy^56–58^. Taken together, our current findings and published studies highlight the role of abnormal growth signaling in different kinds of myopathies. Further studies are required to identify the mechanisms underlying the impact of MYTHO on growth signaling pathways.

**Fig. 8:**
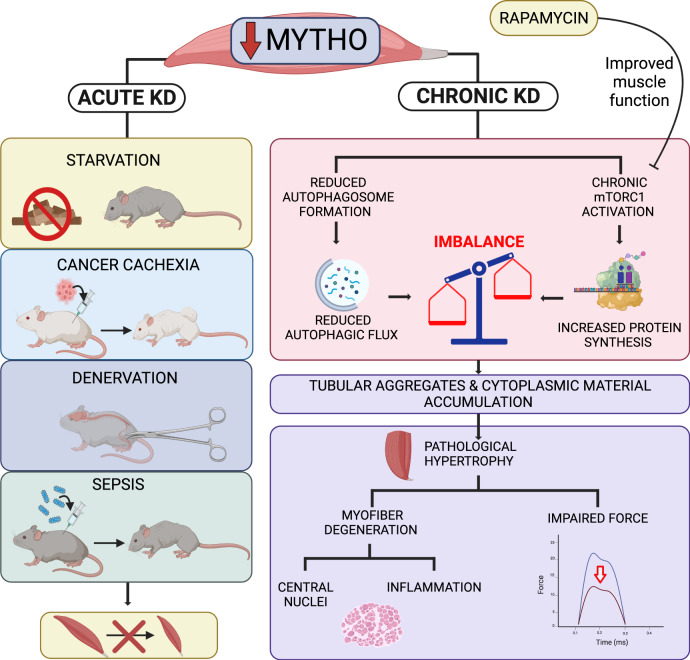
*Mytho*: a novel gene involved in the regulation of autophagy, skeletal muscle mass, and integrity. Scheme illustrating the role of MYTHO in skeletal muscle homeostasis. Acute MYTHO depletion protects from atrophy in different catabolic conditions (starvation, cancer cachexia, denervation, and sepsis). Prolonged MYTHO depletion in skeletal muscle blunts autophagic flux and triggers a sustained hyperactivation of growth signaling causing a myopathological phenotype, characterized by pathological hypertrophy, myofiber degeneration, impaired force generation, and major ultrastructural abnormalities. KD Knockdown. Created with BioRender.com.

In summary, we have identified MYTHO as a novel gene involved in the regulation of autophagy, skeletal muscle mass, and integrity. MYTHO knockdown is indeed sufficient to inhibit basal and starvation-induced autophagy in skeletal muscles. While short-term depletion in MYTHO protects against muscle atrophy in various catabolic conditions, including starvation, cancer cachexia, denervation, and sepsis, its long-term depletion results in a severe myopathic phenotype caused, at least in part, by sustained activation of the mTORC1 pathway (Fig. 8). Whether alterations in MYTHO expression and/or function are implicated in human muscle disorders or other human diseases will require further studies.

## Methods

### Ethic approval for experiments involving animals

All experiments involving animals were approved by the Research Ethics Board of the Research Institute of the McGill University Health Center (MUHC-RI; #2014-7549) or by the committee of the Italian Ministero Salute, Ufficio VI (authorization number 1060/2015 PR). Mice were housed under a standard 12:12-h light/dark cycle, and, unless otherwise indicated, received ad libitum access to a standard chow diet (Teklad global 18% protein irradiated rodent diet 2918, Envigo; or 4RF21, Mucedola, Italy) and water.

### Ethics approval for experiments involving human participants and patients

All procedures were approved by the Ethics Committee of the Université du Québec à Montréal (CIEREH-3477) and the *centre intégré universitaire en santé et services sociaux* of Saguenay‒Lac-St-Jean (2019-002). Patients received a $50 compensation. Informed consent was obtained from all participants. Gender and sex were not considered in the present study due to the limited sample size.

### Generation of skeletal muscle-specific *FoxO1/3/4*^SkM-KO^ and *Atg7*^iSkM-KO^ mice

The generation of skeletal muscle-specific *FoxO1/3/4* triple knockout mice (*FoxO1/3/4*^mKO^)^2^ was performed by crossing mice bearing *FoxO1/3/4*-floxed alleles (*FoxO1/3/4* ^*f/f*^) with transgenic mice expressing Cre under the control of a Myosin Light Chain 1 fast promoter (MLC1f-Cre). Cre-negative littermates were used as controls. We also generated an inducible and muscle-specific *Atg7* knockout mouse model (*Atg7*^iSkM-KO^) by using *Atg7* floxed mice^59^ crossed with mice expressing CreER^T2^ driven by human skeletal Actin (HSA) promoter^60^. Tamoxifen-inducible Cre was activated by a certified rodent diet containing tamoxifen citrate (400 mg/kg) (Teklad TAM400/CreER, Envigo, Madison, WI) for 10 weeks, then returned to a standard chow diet (Teklad global 18% protein irradiated rodent diet 2918, Envigo) for 12 weeks. Littermates, floxed for *Atg7* but not expressing Cre recombinase, were used as controls. All mice were group housed under a standard 12:12-h light/dark cycle with food and water available ad libitum. Mice of the same sex and age were used for each individual experiment.

### Animal models of muscle atrophy

Several experiments were performed to induce muscle atrophy in wild-type C57BL6/J mice, including starvation, denervation, and sepsis. For the starvation experiments, animals were food-deprived for 24 or 48 h before sacrifice early in the morning. Two models of sepsis were used:^61,62^ cecal ligation and puncture (CLP) and intraperitoneal injections of E. coli lipopolysaccharide (LPS) (10 mg/kg of LPS-L2880, Sigma-Aldrich) at 24 or 48 h prior to sacrificed. Denervation was performed by cutting a 5–10 mm long segment of the sciatic nerve from the left limb, while the right limb was used as control. Mice were sacrificed 3-, 7- or 14 days post-surgery. To study cancer cachexia, 7-week-old female BALB/c mice (Charles River Laboratories, Italy) were randomized and divided into two groups: control (mice without tumor inoculation) and C26 (C26-bearing mice).

To study cancer-cachexia, 7-week-old female BALB/c mice (Charles River Laboratories, Italy) were randomized and divided into two groups: control (mice without tumor inoculation) and C26 (C26-bearing mice). C26-derived tumor solid tissue (1-mm^3^) was s.c. implanted using a trocar needle through a small incision in the skin overlying the flank of the mice^63^. At the experimental endpoint, determined by ethical criteria (loss of ~20% of the initial body mass at around 4 weeks after tumor inoculation), muscles were removed and either prepared for histology or frozen in liquid nitrogen and stored at −80 °C for subsequent analyses.

### In vivo electroporation

In vivo electroporation experiments were performed in 3 months old C57BL/6 J female mice (25–28 g) either in the TA or in the flexor digitorum brevis (FDB). Mice that were used for in vivo electroporation experiments were housed under standard 12:12-h light/dark cycle and were fed with a standard chow diet (4RF21, Mucedola, Italy) and water. TA or FDB muscles were exposed through a small incision and plasmids of interest were injected intramuscularly (20 µg of plasmid for the TA and 10 µg for the FDB)^4,64^. Electric pulses were then applied using 2 stainless steel spatula electrodes placed on each side of the isolated muscle belly (TA: 21 V/cm, 20 ms pulse length, and 200 ms pulse interval, for a total of 5 pulses; FDB: 100 V/cm, 20 pulses, 1 s intervals). 7-10 days after electroporation, FDB muscle fibers were isolated enzymatically with collagenase (3 mg/mL) for 1 h 50 min at 37 °C and then mechanically separated. Fibers were then plated in Matrigel overnight at 37 °C. Fibers were then fixed using 4% paraformaldehyde in PBS for 20 min at room temperature for subsequent confocal analysis. One leg was electroporated with control vectors and the other leg with vectors of interest. To silence *Mytho* expression, a *Mytho* sh-RNA vector was generated using Invitrogen BLOCK-iTTM Pol II miR RNAi Expression vector kit. The sequence targeting MYTHO (5’ to 3’) is available in supplementary Table S1. For denervation experiments, sh*Mytho* or shScramble were transfected 5 days before the denervation procedure and for cancer cachexia model the transfection was performed 7 days after tumor inoculation. To overexpress *Mytho*, the coding sequence of the *230025D16Rik gene* (1239 bp) was amplified from cDNA of the cancer cachexia mouse model and cloned in peGFP-N3 vector (4.7 kb) with KOD Hot Start DNA polymerase (Merck Millipore). The primers used for the amplification are detailed in supplementary Table S2.

### LC3-vesicle quantification and colocalization experiments

FDB muscles were co-electroporated with plasmids coding for MYTHO-eGFP and cherry-LC3b or LAMP2-cherry. FDB muscles were isolated and fixed as explained above. Fluorescence images were taken using confocal microscope TCS SP5 LEICA at 63X oil and the quantification was done by counting yellow dots manually and normalizing by the total amount of puncta (green+ red) in the fiber.

### AAV injections in skeletal muscles

Adult (~10-wk-old) male wild-type C57/Bl6j mice (Charles River Laboratories, Saint-Constant, QC) were used for our AAVs experiments. Mice that have received AAV injections were housed under standard 12:12-h light/dark cycle with ad libitum access to food (Teklad global 18% protein irradiated rodent diet 2918, Envigo) and water. All AAVs used in the present study were purchased from Vector BioLabs (Malvern, PA, USA) and were of Serotype 1, a serotype with a proven tropism for skeletal muscle cells^65^. To silence MYTHO, an AAV containing a U6 promoter, a sequence coding for a sh-RNA targeted the product of the 230025D16Rik gene (the gene coding for MYTHO) was injected intramuscularly (i.m.) (25 μl per site; 1.5 × 10^11^ gc) into the right TA and GAS. This AAV is referred to hereafter as AAV sh-RNA Mytho. A control AAV containing a scramble sh-RNA sequence under the control of the U6 promoter was injected into the contralateral leg. This AAV is hereafter referred to as AAV sh-RNA scrmb. To overexpress MYTHO, an AAV containing a tMCK promoter and the sequence coding for MYTHO was injected intramuscularly (25 μl per site; 1.5 × 10^11^ gc) into the right TA and GAS. A control AAV containing the fLuc reporter sequence under the control of the tMCK promoter was injected into the contralateral leg. Injections were carried out under general anesthesia using 2.5 to 3.5% isoflurane. At multiple time points, mice were anesthetized with isoflurane and subsequently euthanized by cervical dislocation. The TA from both legs, was carefully removed and weighed. These samples were then frozen in liquid nitrogen and stored (−80 °C) for immunoblotting and qPCR experiments.

### Autophagic flux quantification

We monitored autophagic flux using colchicine treatment in mice 2 weeks after electroporation or 12 weeks after AAV-mediated transduction. Colchicine (0.4 mg/kg) was administered intraperitoneal (i.p.) twice, at 24 hr and at 12 hr before muscle collection in fed or starved mice.

### Rapamycin administration

Rapamycin is a specific inhibitor of mTORC1, ultimately stimulating autophagy in many cell types, including skeletal muscle. Three weeks after AAVs transfection, WT mice were injected (i.p.) with rapamycin (2 mg/kg/day; LC Laboratories, Woburn, MA, USA) or DMSO (Thermo Fisher Scientific) as vehicle control for 3 weeks. Muscles were then examined after six weeks of MYTHO-KD.

### Transmission electron microscopy

Small strips prepared from the white gastrocnemius fibers were fixed in 2% glutaraldehyde buffer solution in 0.1 M cacodylate, pH 7.4, then post-fixed in 1% osmium tetroxide in 0.1 M cacodylate buffer. Tissues were dehydrated using a gradient of increasing concentrations of methanol to propylene oxide and infiltrated and embedded in EPONTM at the Facility for Electron Microscopy Research at McGill University. Ultrathin longitudinal sections (60 nm) were cut with a Reichert-Jung Ultracut III ultramicrotome (Leica Microsystems), mounted on nickel carbon-formvar coated grids, and stained with uranyl acetate and lead citrate. Sections were imaged using an FEI Tecnai 12 transmission electron microscope at 120 kV and images were digitally captured using an AMT XR80C CCD camera system.

### In situ analysis of muscle contractility

Skeletal muscle contractile function was assessed at various time points after AAV injections using a Dynamic Muscle Data Acquisition and Analysis System (Aurora Scientific, Aurora, ON)^65,66^. To this end, animals were anesthetized with an intraperitoneal injection of a ketamine-xylazine cocktail (ketamine: 130 mg/kg; xylazine: 20 mg/kg). Anesthesia was maintained with 0.05 ml supplemental doses, as needed. A Dynamic Muscle Control and Analysis Software (DMC/DMA) Suite was used for data collection and analysis (Aurora Scientific, Aurora, ON). The distal tendon of the TA muscle was isolated and attached to the arm of a 305C-LR dual-mode muscle lever with 4.0 surgical silk. The partially exposed muscle surface was kept moist and directly stimulated with an electrode placed on the belly of the muscle. Supramaximal stimuli (pulse durations of 2 ms) were delivered using a computer-controlled electrical stimulator (Aurora Scientific, Aurora, ON). Optimal muscle length and voltage were progressively adjusted to produce maximal tension. Force-frequency relationship curves were determined at muscle optimal length at 10, 30, 50, 70, 100, 120, 150, and 200 Hz, with 1 min intervals between stimulations to avoid fatigue. In situ TA force was normalized to the muscle mass as an estimate of specific force. At the end of each experiment, mice were sacrificed, and muscles were carefully dissected, weighed, and frozen in liquid nitrogen or in isopentane pre-cooled in liquid nitrogen for further analyses.

### In situ assessment of mitochondrial function

#### Preparation of permeabilized muscle fibers

Mitochondrial function was assessed in freshly excised GAS muscles. GAS muscles were rapidly dissected and immersed in ice-cold (4 °C) stabilizing buffer A (2.77 mM CaK_2_ EGTA, 7.23 mM K_2_ EGTA, 6.56 mM MgCl_2_, 0.5 mM dithiothreitol (DTT), 50 mM 2‐(N‐morpholino) ethanesulfonic acid potassium salt (K‐MES), 20 mM imidazol, 20 mM taurine, 5.3 mM Na_2_ ATP, and 15 mM phosphocreatine, pH 7.3). Different regions of the GAS muscle have different muscle fiber type compositions and the following protocol was used to minimize variance in results. Mitochondrial respiration and H_2_O_2_ emission were performed in the oxidative red GAS while the assessment of the mitochondrial permeability transition pore sensitivity to Ca^2+^ was assessed in the glycolytic white GAS. GAS samples were weighed and then separated into small fiber bundles using fine forceps and a Leica S4 E surgical dissecting microscope (Leica Microsystems, Wetzlar, Germany). Muscle fiber bundles were incubated for 30 min at low rocking speed in glass scintillation vials containing buffer A supplemented with 0.05 mg/mL saponin to selectively permeabilize the sarcolemma. Red fiber bundles used for respiration and H_2_O_2_ emission analyses were then washed three times for 10 min at low rocking speed in MiR05 buffer (110 mM sucrose, 20 mM HEPES, 10 mM KH_2_PO_4_, 20 mM taurine, 60 mM K-lactobionate, 3 mM MgCl_2_, 0.5 mM EGTA, 1 g/l of fatty acid-free BSA, pH 7.4) at 4 °C. White fiber bundles used for CRC were washed 3 times for 10 min at low rocking speed in buffer C (80 mM K-MES, 50 mM HEPES, 20 mM taurine, 0.5 mM DTT, 10 mM MgCl_2_, and 10 mM ATP, pH 7.3) at 4 °C. Bundles were then transferred into buffer D (800 mM KCl, 50 mM HEPES, 20 mM taurine, 0.5 mM DTT, 10 mM MgCl_2_, and 10 mM ATP, pH 7.3) for 30 min at 4 °C. CRC bundles were then washed three times in a low-EGTA CRC buffer (250 mM sucrose, 10 mM Tris, 5 µM EGTA, and 10 mM 3-(*N-morpholino*) propane sulfonic acid (MOPS, pH 7.3) at 4 °C and kept on ice until measurements were performed.

#### Assessment of mitochondrial respiration

The assessment of mitochondrial respiration in permeabilized TA myofibers was performed using an Oroboros O2K high-resolution fluororespirometer^67^ (Oroboros Instruments; data were recorded with the Data Lab 7.4 software from Oroboros Instruments) at 37 °C in 2 mL of buffer MiR05. Three to 6 mg (wet weight) of TA permeabilized fiber bundles were weighed and added to the respiration chamber. The following substrates were added sequentially: 10 mM glutamate, 5 mM malate (G + M), 2 mM ADP, 10 mM succinate, and 400 μM antimycin A. Respiration rate were normalized as nanomoles of dioxygen per minute per mg of wet muscle mass. All respiration experiments were analyzed with a MitoFun^68^, a homemade code to analyze mitochondrial function data in the Igor Pro 8 software (Wavemetrics, OR, USA).

#### Mitochondrial H_2_O_2_ emission

The H_2_O_2_ production from TA myofiber bundles was assessed by monitoring the rate of H_2_O_2_ release using the Amplex Ultra Red‐horseradish peroxidase (HRP) system^67^. This was performed along with respiration assessment in the Oroboros O2K high-resolution fluororespirometer (Oroboros Instruments) at 37 °C in 2 ml of buffer MiR05 with Amplex Ultra Red (10 μM), SOD (5 U/ml), and HRP (1 U/ml) at 37 °C. A calibration curve was generated daily using successive additions of known [H_2_O_2_] in absence of tissue. H_2_O_2_ emission was normalized as picomoles per minute per milligram of wet muscle mass. All H_2_O_2_ emission experiments were analyzed with MitoFun^68^, a homemade code to analyze mitochondrial function data in the Igor Pro 8 software (Wavemetrics, OR, USA).

#### Mitochondrial Ca2^+^ retention capacity (CRC)

The sensitivity to mitochondrial permeability transition pore (mPTP) opening was evaluated by determining the mitochondrial CRC in the presence of a Ca^2+^ challenge^69^. GAS fiber bundles were placed in a cuvette with 600 μL of CRC assay mix (10 mM Pi, 2.5 mM malate, 5 mM glutamate, 0.5 nM oligomycin, 1 μM calcium-green) at 37 °C. Mitochondrial Ca^2+^uptake was immediately monitored by recording the decrease in extramitochondrial Ca^2+^ concentration using the fluorescent probe calcium-green 5 N (Molecular Probes, Eugene, OR, USA). Fluorescence was detected using a spectrophotometer (Hitachi F2710, FL Solutions software) with excitation and emission wavelengths set at 505 and 535 nm, respectively. Progressive uptake of Ca^2+^ by mitochondria was monitored until mitochondrial Ca^2+^ release occurred due to mPTP opening. CRC was calculated as the total amount of Ca^2+^ taken by mitochondria before Ca^2+^ release. Fluorescent units were converted into Ca^2+^ concentration using a standard curve obtained daily using successive addition of known Ca^2+^ concentration in the absence of sample. CRC values were expressed per milligram of wet fiber weight or per unit of CS activity. All CRC experiments were analyzed with MitoFun^68^, a homemade code to analyze mitochondrial function data in the Igor Pro 8 software (Wavemetrics, OR, USA).

### Histology and immunohistochemistry of muscle cryosections

Muscle samples were mounted in tragacanth (Sigma-Aldrich # G1128) on plastic blocks and frozen in liquid isopentane cooled in liquid nitrogen and stored at −80 °C. Samples were cut into 10 µm cross-sections using a cryostat at −20 °C then mounted on lysine-coated slides (Superfrost). The following histological stains were performed on TA cross-sections: hematoxylin and eosin (H&E), Masson’s trichrome (MT), nicotinamide adenine dinucleotide tetrazolium reductase (NADH-TR), modified Gomori trichrome (MGT) and succinate dehydrogenase (SDH). For all immunohistochemistry experiments except myosin heavy chain immunolabelling, muscle cryosections sections were first allowed to reach room temperature and rehydrated with PBS (pH 7.2) and then fixed in 4% PFA, permeabilized in 0.1% Triton X-100 in PBS for 15 min. Slides were then washed three additional times with PBS and then blocked with goat serum (10% in PBS) for 1 hour. After, muscle sections were incubated with the appropriate primary antibody diluted in 10% goat serum in PBS for 1 hour. Sections were then washed three times in PBS before being incubated for 1 h at room temperature with the appropriate secondary antibodies diluted in 10% goat serum in PBS. After washing, stained sections were mounted with Prolong™ Gold with or without DAPI. All primary and secondary antibodies are listed in Table S3.

To assess muscle fiber type composition, TA muscle sections were immunolabeled for myosin heavy chain (MHC) types I, IIa, and IIb^70^. Muscle cross-sections were first allowed to reach room temperature and rehydrated with phosphate-buffered saline (PBS) (pH 7.2) and then blocked with goat serum (10% in PBS) for 1 h at room temperature. Muscle sections were then incubated for 1 h at room temperature with the following primary antibody cocktail: a mouse IgG2b monoclonal anti-MHC type I (BA-F8, 1:25, DSHB, Iowa, IA, USA), mouse IgG1 mono- clonal anti-MHC type IIa (SC-71, 1:200, DSHB, Iowa, IA, USA), mouse IgM monoclonal anti-MHC type IIb (BF-F3, 1:200, DSHB, Iowa, IA, USA) and a rabbit IgG poly- clonal anti-laminin (Sigma-Aldrich, St Louis, MO, USA, L9393, 1:750). Muscle cross-sections were then washed 3 times with PBS before being incubated for 1 h with the following secondary antibody cocktail: Alexa Fluor 350 IgG2b (y2b), goat anti-mouse antibody (Thermo Fisher Scientific, Waltham, MA, USA, A-21140, 1:500), Alexa Fluor 594 IgG1 (y1) goat anti-mouse (Thermo Fisher Scientific, A-21125, 1:100), Alexa Fluor 488 IgM goat anti-mouse (Thermo Fisher Scientific, A-21042, 1:500) and Alexa Fluor 488 IgG goat anti-rabbit (Thermo Fisher Scientific, A-11008, 1:500). These sections were then washed three times in PBS and coverslipped using Prolong Diamond (P36961; Thermo Fisher Scientific) as mounting medium. Fibers without any staining were considered Type IIx fibers. Images were captured using a Zeiss Axio Imager M2 and the Zen 3.5 software. All MHC-targeting primary antibodies were purchased from the Developmental Studies Hybridoma Bank (DSHB) at the University of Iowa. Given the very low abundance of MHC type I fibers in the TA (<1%), data for these fibers were not included in our analyses. Fiber-type analyses were performed by a single observer blinded to sample identity. Analyses were performed using ImageJ (NIH, Bethesda, Maryland, USA).

### Evans Blue dye uptake quantification

Mice were injected i.p. with 1% (w/v) of Evans Blue dye (EBD) (Sigma-Aldrich #E2129). Mice were euthanized ~24 hrs after EBD injection. EBD excitation was achieved using a 568-nm laser diode, and fluorescence emission was collected at 603 nm.

### In vivo protein synthesis measurements

In vivo protein synthesis in skeletal muscle was measured using the SUnSET technique^30,71^. Mice were weighed and injected i.p. with puromycin dissolved in 100 μl of sterile PBS (0.04 μmol puromycin/g body mass (Sigma-Aldrich # P8833)). At 30 min post puromycin injection, TA muscles were carefully removed and then frozen in liquid nitrogen and stored (−80 °C) for future immunoblotting analysis using a mouse IgG2a monoclonal anti-puromycin antibody (clone 12D10, 1:2500; Millipore # MABE343).

### Immunoblotting

Frozen skeletal muscle tissues (~15 mg) were homogenized in an ice-cold lysis buffer A (50 mM Hepes, 150 mM NaCl, 100 mM NaF, 5 mM EDTA, 0.5% Triton X-100, 0.1 mM DTT, 2 µg/ml leupeptin, 100 µg/ml PMSF, 2 µg/ml aprotinin, and 1 mg/100 ml pepstatin A, pH 7.2) or Lysis Buffer B (50 mM Tris pH 7.5, 150 mM NaCl, 10 mM MgCl2, 0.5 mM DTT, 5 mM EDTA, 10% glycerol, 2%SDS, 1%Triton X-100, protease Inhibitor cocktail and phosphatase inhibitors cocktail I and II (Roche)) using Mini-beadbeater (BioSpec Products) with ceramic bead at 60 Hz. Muscle homogenates were kept on ice for 30 min with periodic agitation and then were centrifuged at 5000 g or 14,000 × *g* for 15 min at 4 °C, supernatants were collected, and pellets were discarded. The protein content in each sample was determined using the Bradford or BCA (Pierce) method. Aliquots of crude muscle homogenate were mixed with Laemmli buffer (6×, reducing buffer, # BP111R, Boston BioProducts) and subsequently denatured for 5 min at 95 °C. Equal amounts of protein extracts (30 µg per lanes) were separated by SDS-PAGE, and then transferred onto polyvinylidene difluoride (PVDF) or nitrocellulose membranes (Bio-Rad Laboratories) using a wet transfer technique. The total proteins on membranes were detected with Ponceau-S solution (Sigma #P3504) or the stain free technology from Bio-Rad. Membranes were blocked in PBS + 1% Tween® 20 + 5% bovine serum albumin (BSA) or TBS + 1% Tween® 20 + 5% milk for 1 hour at room temperature and then incubated with the specific primary antibodies overnight at 4 °C. The complete list of antibodies used for immunoblots analyses can be found in Supplementary Table S4. Membranes were washed in PBST or TBST (3×5 min) and incubated with HRP-conjugated secondary anti-rabbit or anti-mouse secondary antibodies (Abcam, cat# Ab6728, Ab6721) for 1 hour at room temperature, before further washing in PBST (3 × 5 min). Immunoreactivity was detected using enhanced chemiluminescence substrate (Pierce™, Thermo Fisher Scientific) with the ChemiDoc™ Imaging System. The optical densities (OD) of protein bands were quantified using ImageLab 6.1 software (Bio-Rad Laboratories) and normalized to loading control. Immunoblotting data are expressed as relative to Ctrl.

### Quantitative real-time PCR

Total RNA was extracted from frozen muscle samples using PureLink™ RNA Mini Kit (Invitrogen Canada, Burlington, ON) or Trizol (Life Technologies) following the manufacturer’s instructions. Quantification and purity of RNA were assessed using the A260/A280 absorption method. Total RNA (2 μg or 400 ng) was reverse transcribed using a Superscript II® Reverse Transcriptase Kit and random primers (Invitrogen Canada, Burlington, ON) or the SupersCript IV (Life technologies). Real‐time PCR detection of mRNA expression was performed using a Prism® 7000 Sequence Detection System (Applied Biosystems, Foster City, CA) or a QuantStudio 5 Real-Time PCR System (Thermo Fisher Scientific, Waltham, MA) with SYBR Green chemistry (Power-UP SYBR®Green PCR Master Mix, Applied Biosystem). Cycle threshold (CT) values were obtained for each target gene. ΔCT values (normalized gene expression) were calculated as CT of the target gene minus CT of the geometric means of three housekeeping genes (*Cyclophilin*, *Gapdh*, *β-Actin,* and/or *18* *S*), unless otherwise indicated in the figure legends. Relative mRNA level quantifications of target genes were determined using the threshold cycle (ΔΔCT) method, as compared to Ctrl. The primer sequences for all genes are found in Supplementary Table S5.

### Human muscle biopsy specimens

Skeletal muscle biopsy samples were obtained from the *vastus lateralis* muscle using a suction-modified Bergström needle performed under local anesthesia. *Mytho* RT-PCR analysis was performed on eight DM1 samples (5 females, 3 males; age: 51.8 ± 4.0; CTG repeats: 476 ± 59) and 11 healthy samples (5 females, 6 males; age 45.8 ± 6.7). Immunoblotting analyses were performed on 6 DM1 samples (6 males; age: 58,5 ± 2.5; CTG repeats: 233.5 ± 74.68) and 8 healthy samples (8 males; age: 64.7 ± 2.7).

### Microarrays

Total RNA was isolated from GAS muscle samples (20–25 mg) that were obtained at 3 and 12 weeks post AAV injections. A total of 21,981 mouse genes were included in the Affymetrix Mouse Clariom S Assay (Affymetrix, Santa Carla, CA) and all steps were performed at the McGill University and Génome Québec Innovation Center (Montréal, QC). Raw data were analyzed using default parameters from the Transcriptome Analysis Console (TAC) 4.0.1 software (Affymetrix) using default parameters. The ANOVA method (ebayes) was used to identify expression level differences between AAV sh-RNA MYTHO and AAV sh-RNA scramble muscles with at least a twofold change (*p* < 0.05). Additionally, false discovery rate (FDR) calculations to correct for multiple testing were applied according to Benjamini and Hochberg^72^.

### Bioinformatic and gene ontology analyses

Gene Ontology analysis was performed on all significantly up- and downregulated genes upon MYTHO knockdown (161 and 53 genes, respectively), using Metascape (http://metascape.org). The gene-level signal intensities after SST-RMA normalization were used to generated heatmaps and hierarchical clustering using webtool Clustvis^73^. FunRich software 3.1.4^74^ was used to generate a Venn diagram of upregulated genes obtained from the NCBI Gene Expression Omnibus (GEO): GSE48363 (GAS/plantaris muscle/cancer)^75^, GSE63032 (GAS muscle/cancer cachexia)^76^ and GSE20103 (TA muscle /24 h STV)^77^. For microarray data set, GEOexplorer (https://geoexplorer.rosalind.kcl.ac.uk)^78^, was utilized to identify differentially expressed genes (adj *p* < 0.05 and fold change >1.25) across experimental conditions. We also analyzed *Mytho* (aka D230025D16Rik) mRNA levels (FDR < 0.05 and fold change >1.25) in GAS muscle of 8 and 28 months old wild-type mice (GSE145480) using SarcoAtlas (https://sarcoatlas.scicore.unibas.ch/)^79^.

The RNA-seq data of TA muscles from healthy and myotonic dystrophy type 1 (DM1, aka Steinert disease) individuals were acquired from the Myotonic Dystrophy Deep Sequencing Data Repository (http://www.dmseq.org/) and GEO (GSE86356) repositories, respectively. DM1 patients were classified as mild, moderate, or severe^80^. *Mytho* (aka C16ORF70) mRNA levels (transcripts per million, TPM) were analyzed in tibialis anterior muscle biopsies from control or DM1 patients using one-way ANOVA followed by two-stage linear step-up procedure of Benjamini, Krieger, and Yekutieli (*p* < 0.05 and *q* < 0.1 was considered statistically significant).

### Statistical analyses

Unless otherwise indicated, comparisons between two independent groups were performed with unpaired two-tailed *t* tests while comparisons between two related groups were performed using paired two-tailed *t* tests. Comparisons between more than two groups were performed using either one-way or two-way Analysis of variance (ANOVA). Corrections for multiple comparisons were performed using the two-stage step-up method of Benjamini, Krieger, and Yekutieli (*p* < 0.05 and *q* < 0.1 were considered significant) All statistical analyses were performed using GraphPad Prism 9.4.0.

### Reporting summary

Further information on research design is available in the Nature Portfolio Reporting Summary linked to this article.

## Supplementary information

Supplementary Information

Reporting Summary

## Data Availability

The datasets generated during and/or analyzed during the current study are available from the corresponding authors on request. Source data are provided with this paper. Microarray data generated in this study have been deposited in the Gene Expression Omnibus (GEO) database under accession codes GSE218706 and GSE222884. The following publicly available GEO datasets were analyzed in the present study: GSE63032, GSE20103, GSE48363, GSE145480 and GSE86356. Source data are provided with this paper.

## Source data

Source Data

## Code availability

MitoFun^68^, the code generated to analyze mitochondrial respiration, H_2_O_2_ emission, and calcium retention capacity using the Igor Pro 8 software (Wavemetrics, OR, USA), is available on Zenodo (10.5281/zenodo.7510439).
