# Supplementary material for: MYTHO is a novel regulator of skeletal muscle autophagy and integrity
Source: Nat Commun. 2023 Mar 2;14:1199. doi: 10.1038/s41467-023-36817-1 (PMC9981687; doi:10.1038/s41467-023-36817-1)
Supplement: Supplementary file 1 — Supplementary Information [file 41467_2023_36817_MOESM1_ESM.pdf]

MYTHO IS A NOVEL REGULATOR OF SKELETAL MUSCLE AUTOPHAGY AND INTEGRITY

Supplementary figures and legends

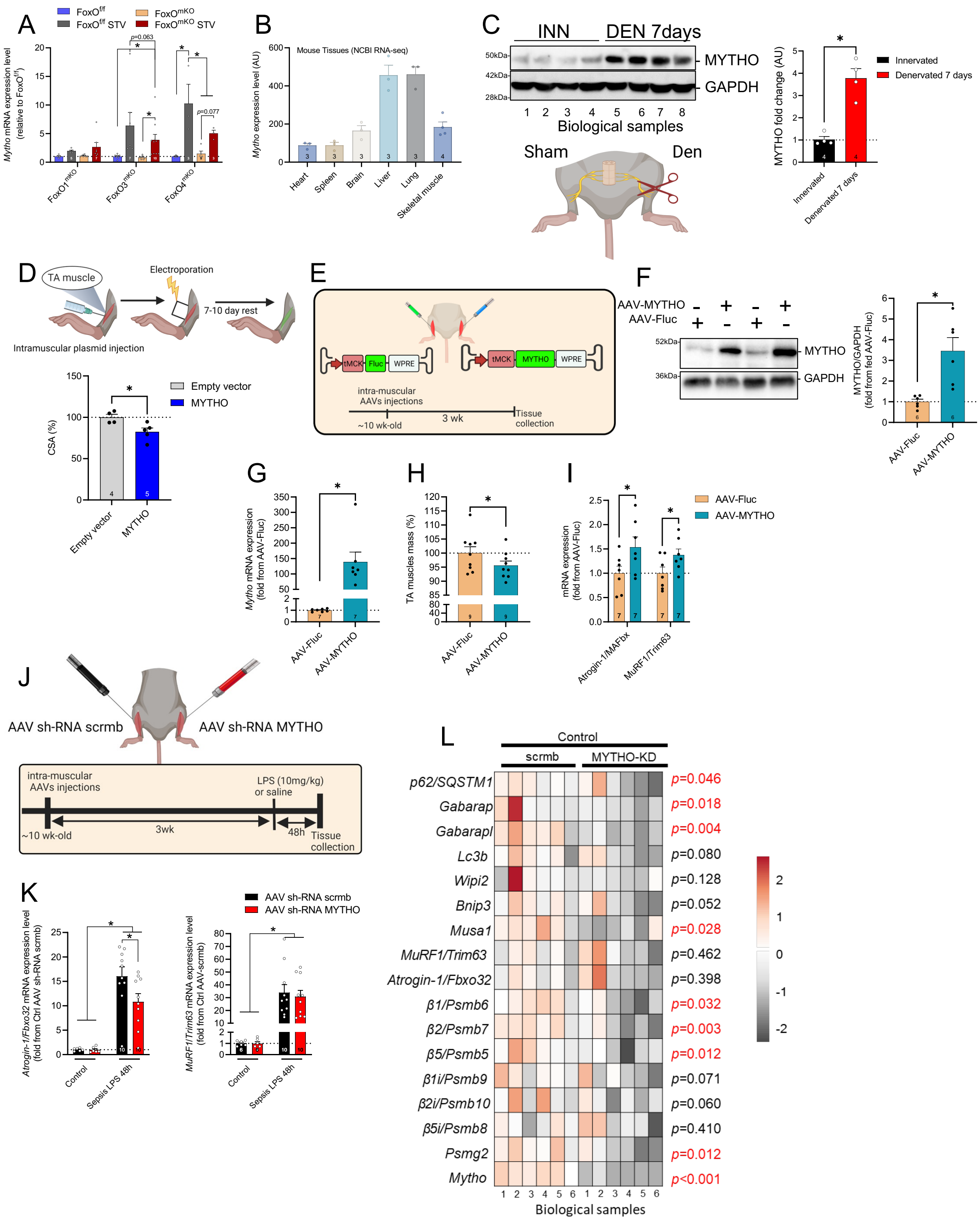

Figure S1: Mytho is expressed in various tissues and is upregulated in catabolic conditions. A Quantification of *Mytho* mRNA levels assessed using RT-qPCR in the TA of fed and 24-h starved FoxO1$^{f/f}$, FoxO3$^{f/f}$, FoxO4$^{f/f}$,

FoxO1$^{-/- mKO}$, FoxO3$^{-/- mKO}$ and FoxO4$^{-/- mKO}$ mice. Results are shown as fold increase from FoxO$^{f/f}$ Fed. GAPDH was used as houseke20eping gene. **B** Microarray analysis from a publicly available GEO data set (accession number: GSE24207) showing *Mytho* expression in various tissues. **C** Immunoblot analysis of MYTHO from homogenates of denervated or innervated TA muscles. GAPDH was used as loading control. All values are expressed relative to innervated TA muscles. **D** Adult TA muscles were transfected with pBI-GFP (one leg) or pBI-GFP 3xflag MYTHO (contralateral leg). After 7 to 10 days, cross-sectional area of transfected fibers was quantified. **E** Schematic representation of the experimental design: TA muscles were transduced with AAV-Fluc and AAV-MYTHO and muscles were examined 3 weeks later in Fed or 48h starved mice. **F-G** MYTHO protein content (**F**) and mRNA expression (**G**) were quantified by immunobloting and RT–qPCR , respectively. **H** Analysis of TA muscle mass (shown as % of AAV-fluc)**. I** Quantification of *Murf1* and *Atrogin1* gene expression by RT-qPCR. Data is shown as fold increase from AAV fluc. **J** Schematic representation of the experimental design. TA muscles were transfected with either AAV sh-RNA scramble or AAV sh-RNA MYTHO. Transduced muscles were examined 3 weeks later. **K** *Murf1* and *Atrogin1* mRNA expression levels were assessed by RT-qPCR in TA samples from control and septic (LPS-injected) mice. Data is shown as fold-increase from AAV sh-RNA scramble. **L** Heatmap showing mRNA expression levels of genes regulating catabolic signaling in TA muscles 3 weeks post transfection as determined by RT-qPCR. Colors indicate relative expression levels; red indicates high expression and gray indicates low expression. Data are presented as mean ± SEM (with individual values) and the number of independent biological replicates is indicated within bars. Data in **A** and **K** were analyzed with two-way ANOVA and corrections for multiple comparisions were performed with the two-stage step-up method of Benjamini, Krieger and Yekutieli ($*p < 0.05$ and $q<0.1$). Comparisons in **C**, **D**, **F**, **G**, **H**, **I** were performed using paired two-tailed t-test ($*p<0.05$). Comparisons in **L** were performed using paired one-tailed t-tests ($*p < 0.05$). Detailed information on raw data, statistical tests, p-values and q-values are provided in the Source Data file. The drawings in **C, D, E** and **J** were created with BioRender.com.

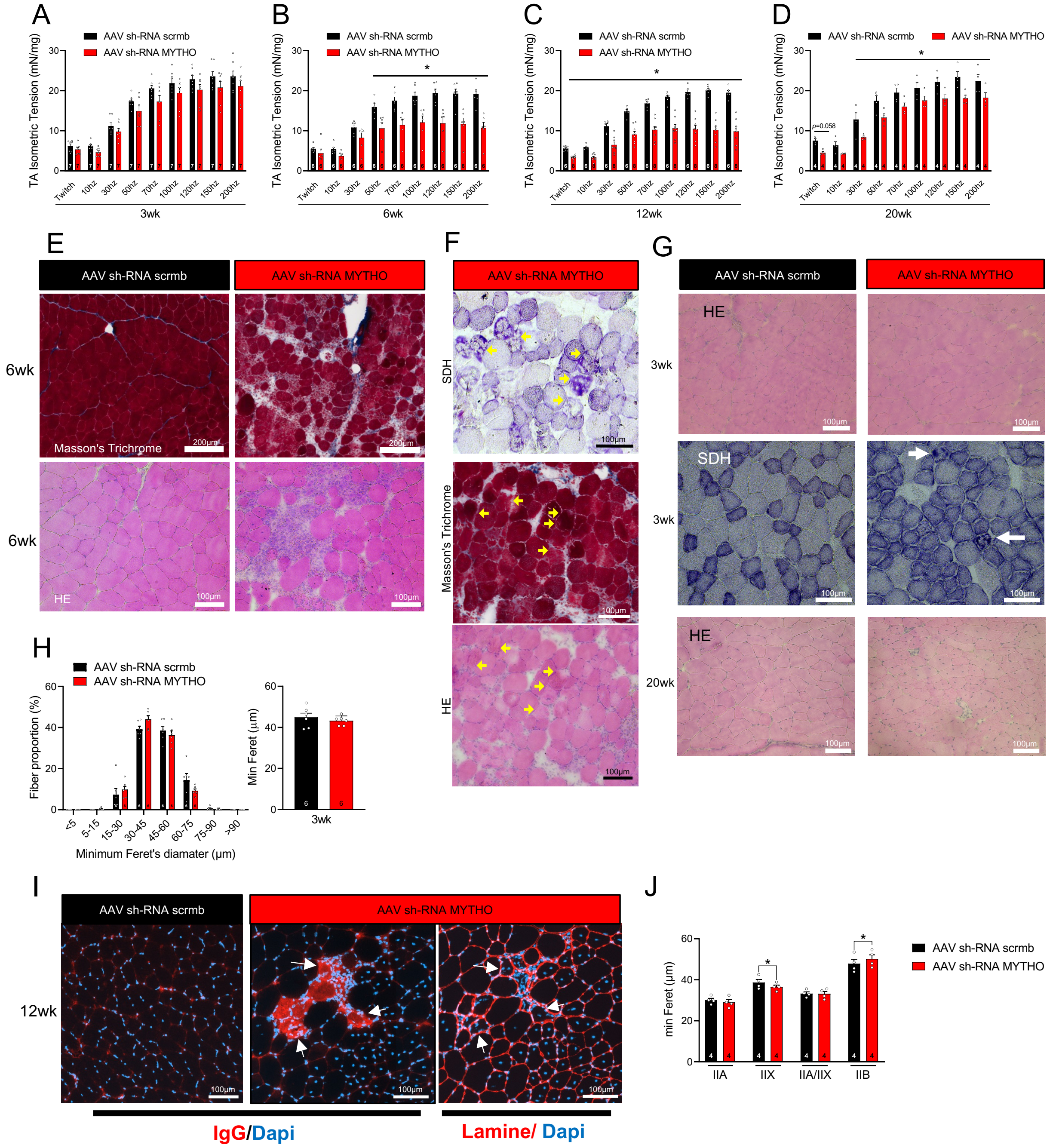

Figure S2. MYTHO regulates muscle mass, fiber-type composition and anabolic signaling in mice. A-D TA isomeric tension measured *in situ* at 3, 6, 12 and 20 weeks post injection of AAV sh-RNA scramble or AAV sh-RNA MYTHO (data were analysed with two-way ANOVA with Benjamini, Krieger and Yekutieli multiple comparisons test: ∗=p < 0.05 and q<0.1). **E** Representative images of Masson's trichrome staining (upper pannel) and H&E staining (lower pannel) at 6 weeks post injection of AAV sh-RNA scramble or AAV sh-RNA MYTHO (n=6 mice per group). Scale bars: 200 µm for upper pannel, 100µm for lower pannel. **F** SDH, Masson's trichrome and H&E staining of the same muscle area at 6 weeks AAV posttransduction (n=6 mice per group). Yellow arrows indicate myofiber abnormalities (ragged blue fibers, miofiber necrosis and small regenerating fibers). Scale bars:

100 µm. **G** Representative images of H&E and SDH staining at 3 weeks (n=6 mice per group) or 20 weeks (n=4 mice per group) post injection of AAV shRNA scramble or MYTHO. White arrows indicate rare abnormalities in myofibers at 3 weeks postransduction. Scale bar: 50µm. **H** Analysis of fiber diametre in TA injected for 3 weeks with either AAV sh-RNA scramble or AAV sh-RNA MYTHO**.** Data in the grah on the left were analysed withtwo-way ANOVA with Benjamini, Krieger and Yekutieli multiple comparisons test (∗=p < 0.05 and q<0.1). Data in the grah on the right were analysed with a paired two-tailed t-test (*p<0.05). **I** Representative images of IgG, laminin and DAPI immunostaining of TA at 3 weeks post injection of AAV sh-RNA scramble or AAV-shRNA MYTHO (n=8 mice per group). White arrows indicate necrotic myofibers. Scale bars: 100 µm. **J** Analysis of fiber type proportion in TA injected with either AAV sh-RNA scramble or AAV sh-RNA MYTHO for 12 weeks. (data were analysed with two-way ANOVA with Benjamini, Krieger and Yekutieli multiple comparisons test: ∗=p < 0.05 and q<0.1). The number of mice for each group is indicated within bars. Data are presented as mean ± SEM (with individual data). ∗=p<0.05 and q<0.1 when applicable. Detailed information on raw data, statistical tests, p-values and q-values are provided in the Source Data file.

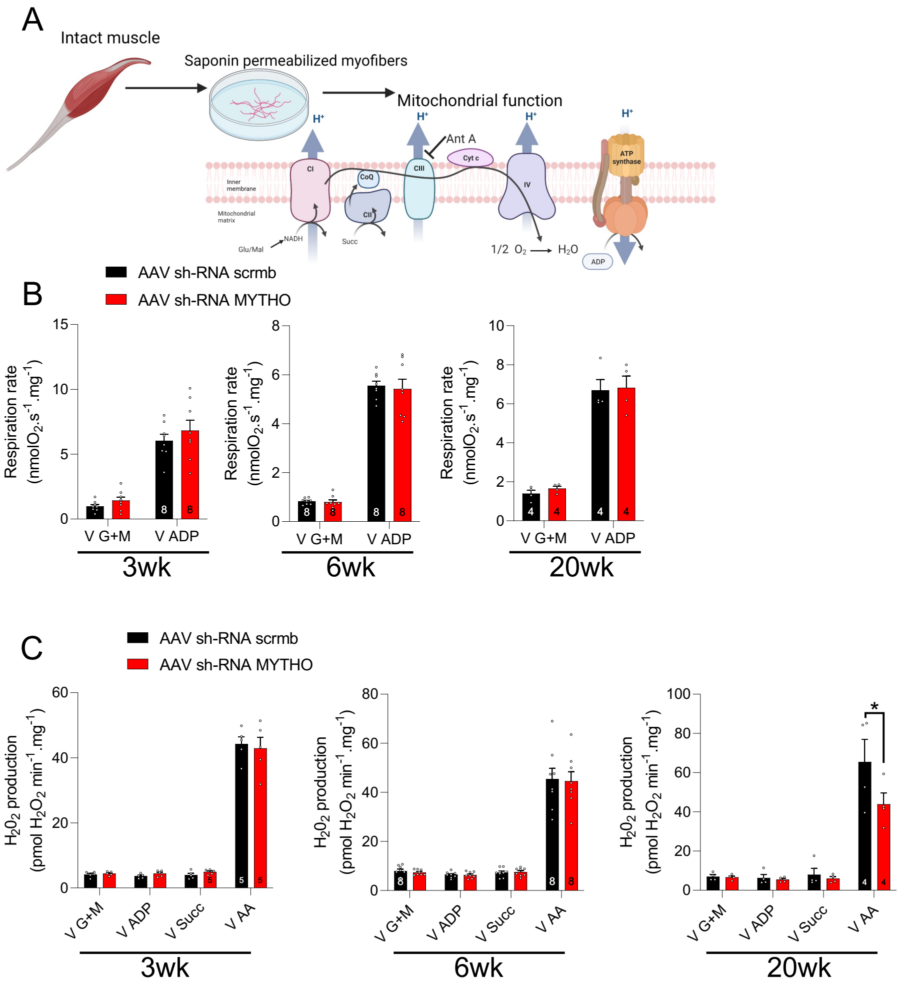

Fig S3. MYTHO depletion does not impair mitochondrial respiration. A Schematic representation of the experimental design. **B** Mitochondrial respiration analysis of GAS muscles transduced for 3, 6 or 20 weeks with AAV sh-RNA scramble or AAV sh-RNA MYTHO. V G+M: respiration rate driven by the addition of Glutamate and Malate. V ADP = respiration rate driven by the subsequent addition of ADP. **C** H_2O_2 emission at 3, 6 and 20 weeks in muscles injected with AAV sh-RNA scramble or AAV sh-RNA MYTHO. V G+M: H_2O_2 production rate driven by the addition of Glutamate and Malate. V ADP = H_2O_2 production rate driven by the subsequent addition of ADP. V Succ: H_2O_2 production rate driven by the subsequent addition of Succinate. V AA: H_2O_2 production rate driven by the subsequent addition of Antimycin A. The number of mice for each group is indicated within bars. Data in **B** and **C** were analysed with two-way repeated measure ANOVA with Benjamini, Krieger and Yekutieli multiple comparisons test (*=$p<0.05$ and $q<0.1$). Data are presented as mean ± SEM (with individual data). Detailed information on raw data, statistical tests, p-values and q-values are provided in the Source Data file. **A** was created with BioRender.com.

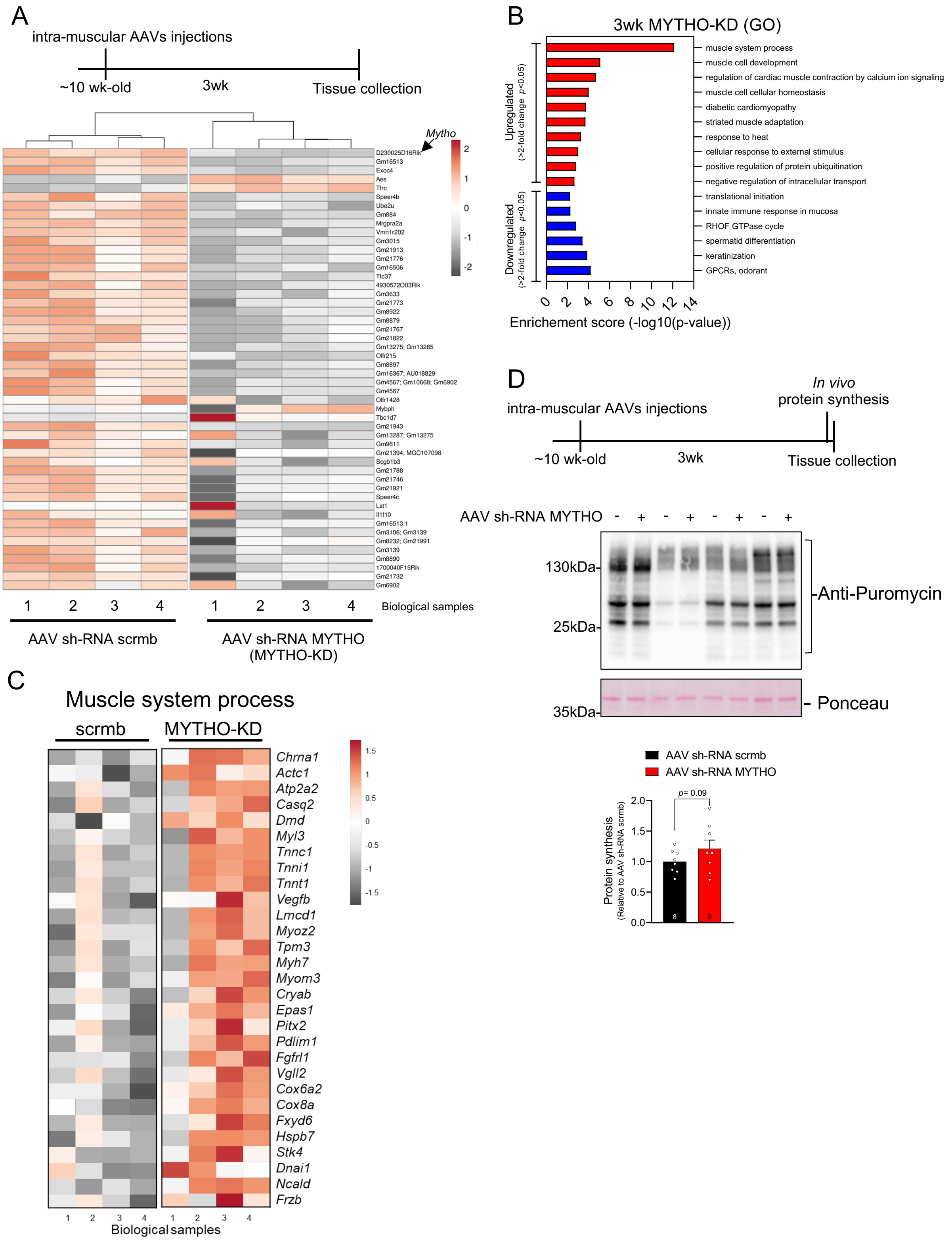

Figure S4: MYTHO depletion activates growth signaling. A Heatmap of the top 50 most robustly regulated genes (>2-fold change and p < 0.05 and q<0.1) derived from microarray analysis of Gastrocnemius (GAS) muscle at 3 weeks post AAV sh-RNA scramble or AAV sh-RNA MYTHO injections. Colors indicate relative

expression levels; red indicates high expression and gray indicates low expression. **B** Top upregulated (red) and downregulated (blue) pathways as identified through GO enrichment analysis. **C** Heatmap of differentially expressed genes extracted from the GO annotation muscle system process. Upregulated genes in GAS injected with AAV sh-RNA MYTHO appear in red while downregulated genes appear in gray. **D** Representative immunoblot and corresponding quantification of puromycin incorporation in TA from AAV sh-RNA scramble and AAV sh-RNA MYTHO injected muscles at 3 weeks post transduction. Data are presented as fold-increase from AAV sh-RNA MYTHO. The number of mice for each group is indicated within bars. Data in **D** were analysed with a paired one tailed *t*-test. Data are presented as mean ± SEM (with individual data). Detailed information on raw data, statistical tests, p-values and q-values are provided in the Source Data file.

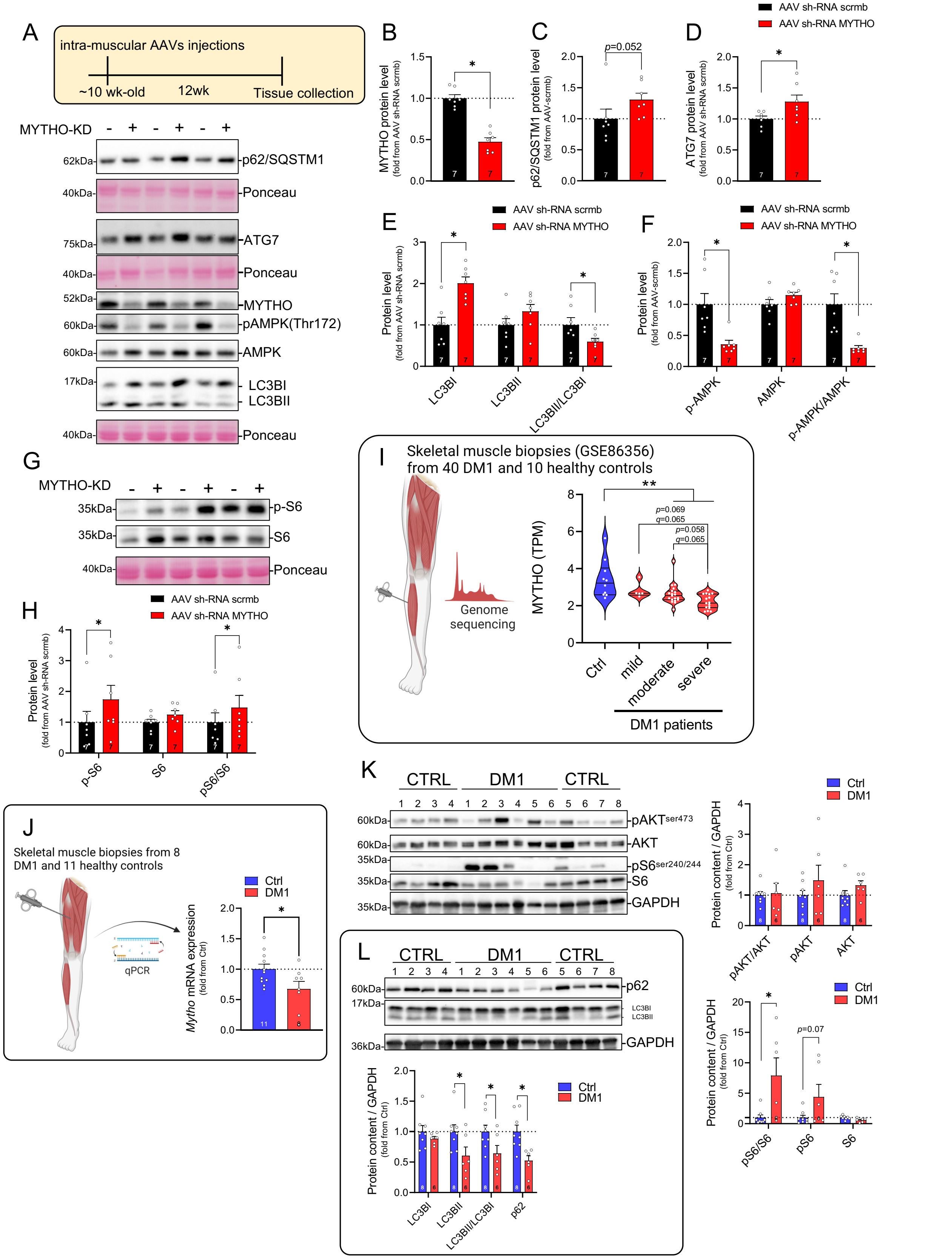

Figure S5: MYTHO depletion activates growth signaling. A-F Immunoblots performed on TA homogenates 20 weeks after the injection of AAV sh-RNA scramble or AAV sh-RNA MYTHO.

Quantification of MYTHO, p62, ATG7, LC3BI, LC3II, LC3II/ LC3I, pAMPK, AMPK and pAMPK/AMPK are shown in graphs **D** to **H**. Results are presented as fold increase from AAV sh-RNA scramble. **G-H** Immunoblots and corresponding quantifications of p-S6 and S6 content in TA homogenates transduced with AAV sh-RNA scramble or AAV sh-RNA MYTHO for 20 weeks. **I** *Mytho* transcript per million (TPM) in TA muscle from patients with mild, moderate and severe myotonic dystrophy type 1 (DM1) (GSE86356). **J** Quantification of *Mytho* gene expression by RT-qPCR in the *vastus lateralis* of patients with DM1 (5 females, 3 males; age: 51.8±4.0; CTG repeats: 476±59), compared to healthy samples (5 females, 6 males; age: 45.8±6.7). **K-L** Immunoblots and corresponding quantifications of pAKT, AKT, pS6, S6 and GAPDH in *vastus lateralis* homogenates of patients with DM1 (6 males; age 58.5±2.5; CTG repeats: 233.5±74.68) and healthy controls (8 males; age: 64.7±2.7). The number of participants/patients or mice for each group is indicated within bars. Data are presented as mean ± SEM (with individual data). ∗=$p < 0.05$ and $q<0.1$ when applicable. Detailed information on raw data, statistical tests, p-values and q-values are provided in the Source Data file. The drawings in **A**, **I** and **J** were created with BioRender.com.

Supplementary tables.

Table S1: shRNA sequence used to downregulate *Mytho* expression.

Gene	Targeting sequence (5'-3')
D230025D16Rik (Mytho) ***shRNA***	TGCTGTAAGGATGAGGTCATGGCTTAGT<br>TTTGGCCACTGACTGACTAAGCCATCCTCATCCTTA

Table S2: Primers used for *Mytho* cloning.

Gene	Forward primer (5'-3')	Reverse primer (3'-5')
D230025D16Rik (Mytho)	AAAGCTAGCATGCTGGACCTGGAGGTGGT	TAAGGATCCGGGCAGCTCTGCTGTTC

Table S3: Antibodies used for *in situ* immunolabeling.

Antibody	Source / Product no.	Dilution	Analysis
Mouse IgG2b monoclonal anti-MHC type I	DSHB #BA-F8	1:25	IF
Mouse IgG1 mono- clonal anti-MHC type IIa	DSHB #SC-71	1:200	IF
Mouse IgM monoclonal anti-MHC type IIb	DSHB #BF-F3	1:200	IF
Rabbit polyclonal anti-laminin	Sigma-Aldrich # L9393	1:750	IF
Mouse monoclonal anti-Stim1	BD Biosciences # 610954	1:200	IF
Mouse monoclonal anti-Serca2	ThermoFisher, MA3-910	1:200	IF
Rat monoclonal 4/80 - Macrophage Marker	Abcam #ab6640	1:100	IF
Alexa Fluor 350 IgG2b (y2b) goat anti-mouse	Invitrogen, A-21140	1:500	IF
Alexa Fluor 488 IgG goat anti-rabbit	Invitrogen, A-11008	1:500	IF
Alexa Fluor 488 IgM goat anti-mouse	Invitrogen, A-21042	1:500	IF
Alexa Fluor 594 IgG1 (y1) goat anti-mouse	Invitrogen, A-21125	1:100	IF
Alexa Fluor 568 IgG goat anti-rat	Invitrogen, A-11007	1:500	IF
Alexa Fluor 594 IgG goat anti-rabbit	Invitrogen, A-11037,	1:500	IF
Alexa Fluor 568 IgG goat anti-rabbit	ThermoFisher, A-11011	1:500	IF

MHC: Myosin heavy chain; DSHB: Developmental Studies Hybridoma Bank (University of Iowa, IA).

Table S4: Antibodies used for immunoblotting.

Antibody	Source / Product no.	Dilution	Analysis
Rabbit anti-Atg7	Cell signaling #8558	1/1000	WB
AKT	Cell signaling #9272	1/1000	WB
p-AKT (Ser473)	Cell signaling #9271	1/1000	WB
Rabbit anti-phospho AMPKα (Thr172)	Cell signaling #2535	1/1000	WB
Rabbit anti-AMPKα	Cell signaling #2532	1/1000	WB
Rabbit anti-LC3	Cell signaling #12741	1/1000	WB
Rabbit anti-LC3B	Sigma #L7543	1/1000	WB
Rabbit anti-GAPDH	Cell signaling # 2118	1/2500	WB
Mouse anti-GAPDH	Santa Cruz #32233	1/10000	WB
Mouse anti-Bnip3	Sigma-Aldrich #B7931	1/1000	WB
Anti-puromycin, clone 12D10	Millipore # MABE343	1/2500	WB
Mouse anti-p62/SQSTM1	Novus Biologicals Inc. clone 2C11	1/1000	WB
Rabbit anti-C16orf70 (MYTHO)	Abcam # 181987	1/1000	WB
Mouse anti-Desmin	D76 Developmental Studies Hybridoma Bank (DSHB)	1/500	WB
Rabbit anti-phospho-S6 (Ser240/244)	Cell signaling #2215	1/1000	WB
Rabbit anti-total S6	Cell signaling #2217	1/1000	WB
Goat anti mouse IgG	Abcam # Ab6728	1/5000	WB
Goat anti rabbit IgG	Abcam # Ab6721	1/5000	WB

Further details about the validation of those antibodies above can be found in the Reporting Summary.

Table S5: Primers used for qPCR.

Gene	Forward primer (5'-3')	Reverse primer (3'-5')
	**Mouse primers sequences**	
D230025D16Rik (Mytho) Isoform201/203	CGCTCCTACCATTGAGCAAA	CCTCGGAAGTTGAGGTGGAA
Lc3b	CGATACAAGGGGGAGAAGCA	ACTTCGGAGATGGGAGTGGA
p62/Sqstm1	GCACCTGTCTGAGGGCTTCT	GCTCCAGTTTCCTGGTGGAC
Bnip3	TTCCACTAGCACCTTCTGATGA	GAACACCGCATTTACAGAACAA
Gabarapl1	GAGGACCACCCCTTCG	CGGAGGGCACAAGGTACTTC
Gabarap	TTCTTGATCCGGAAGCGAAT	CTGGTACAGCTGACCCATCG
Wipi2	TTGATGCAAGTGGGACCAAG	GGAGCAGATGCTCACACACC
Musa1/ Fbxo30	TCGTGGAATGGTAATCTTGC	CCTCCCGTTTCTCTATCACG
MAFbx/Atrogin-1/ Fbxo32	TGGGTGTATCGGATGGAGAC	TCAGCCTCTGCATGATGTTC
MuRF-1/Trim63	TGCTTGGCACTTGAGAGGAA	AGAAGCTGGGCTTCATCGAG
β5/Psmb5	GTACAAAGGCATGGGGCTGT	CGGTCCCAGAGATCCTGTTC
β1/Psmb6	GCAGTTCACTGCCAATGCTC	CAACGTGGCAATGGTGAACT
β2/Psmb7	TTGTCGCAGGAATGCTGTCT	CAGCAACAACCATCCCTTCA
β5i/Psmb8	TACCTGCTTGGCACCATGTC	CGTTCCCCATTCCGAAGATA
β1i/Psmb9	GGACGGAAGAAGTCCACACC	GTGCAGAGGGGAGAGCTTGT
β2i/Psmb10	GCTGCGGACACTGAGATGAC	TTGGTACCGGAAAAGCGTCT
Psmg2	AGCTGCGCAGTACTCCCTTC	ATCTCAGGGATGCACCGACT
MyoG	GCACTGGAGTTCGGTCCCAA	TATCCTCCACCGTGATGCTG
Myh1	TTCCTCCTTCCAGACCGTGT	AGGACCAGTTCGTGCTCCAT
Myh2	ACTTTGGCACTACGGGGAAAC	CAGCAGCATTTCGATCAGCTC
Myh4	CTTTGCTTACGTCAGTCAAGGT	AGCGCCTGTGAGCTTGTAAA
Myh8	AAGAACCCAGGCGGTCTGTA	CGCGGACGTTGTACTGGATA

Cathepsin L	CGGGTTGCCTAGAAGGACAG	ACAGCCCTGATTGCCTTGAT
18S	TGCGGTTTAGCGTCGGTGTC	CCAAGTGGCCAAAGCGTA
β-Actin	AACCGTGAAAAGATGACCCAG	CACAGCCTGGATGGCTACGTA
Cyclophilin	GCGTCTCTTCGAGCTGTTT	CTGGCACATGAATCCTGGAA
Gapdh	AAGAAGGTGGTGAAGCAGGCG	ACCAGGAAATGAGCTTGACAA
	**Human primer sequences**	
D230025D16Rik (Mytho)	TGGGCAATGTCTATGCTGAG	CTTTGTGTGGAGAGCCAAGC
Cyclophilin	CATACGGGTCCTGGCATC TT	AACACCACATGCTTGCCATC

Table S6: Software and Algorithms

**Software and Algorithms**	**Source**	**Identifier**
GraphPad Prism 9.4.0	N/A	https://www.graphpad.com/scientific-software/prism/
Igor Pro (Version 8)	N/A	WaveMetrics
(Fiji) ImageJ	N/A	https://fiji.sc/
BioRender	N/A	BioRender.com
Affymetrix® Transcriptome Analysis Console (TAC) 4.0.1	N/A	Thermo Fisher Scientific
Metascape	N/A	https://metascape.org/gp/index.html#/main/step1
ClustVis	N/A	https://biit.cs.ut.ee/clustvis/
ImageLab	N/A	Bio-Rad Laboratories
Zeiss ZEN 3.5 image acquisition software	N/A	Carl Zeiss
Funrich software	N/A	http://www.funrich.org
Dynamic Muscle Control and Analysis Software	N/A	Aurora Scientific Inc
GEOexplorer	N/A	https://geoexplorer.rosalind.kcl.ac.uk
Mitofun	N/A	https://zenodo.org/record/7510439#.Y9COzi3pOXI
